# Sigma-1 receptor alleviates postoperative cognitive dysfunction exacerbated by preoperative acute sleep deprivation in mice via modulating the Keap1/Nrf2 signaling pathway

**DOI:** 10.1016/j.neurot.2026.e01109

**Published:** 2026-10-29

**Authors:** Xiuhua Li, Baorui Guo, Jiatai An, Yuansong Wei, Jin Yu, Peng Li, Aiping Zheng, Liang Xu, Yalan Li

**Affiliations:** a Department of Anesthesiology, The First Affiliated Hospital of Jinan University, Guangzhou, Guangdong Province, 510642, China; b Academy of Military Medical Sciences, Beijing, 100850, China; c Senior Department of Neurosurgery, Chinese PLA General Hospital, Beijing, 100853, China; d Senior Department of Neurology, Chinese PLA General Hospital, Beijing, 100853, China; e Senior Department of Anesthesiology, Chinese PLA General Hospital, Beijing, 100048, China

**Keywords:** Sleep deprivation, Postoperative cognitive dysfunction, Sigma-1 receptor, Keap1/Nrf2

## Abstract

Postoperative cognitive dysfunction (POCD) is a common neurological complication severely affecting patient prognosis. Preoperative acute sleep deprivation, a frequent perioperative stressor, significantly exacerbates POCD severity. Sigma-1 receptor (σ-1R), an endoplasmic reticulum molecular chaperone, plays a crucial role in modulating neuroinflammation and oxidative stress. However, whether σ-1R can serve as an interventional target to mitigate sleep deprivation-aggravated cognitive impairment remains insufficiently elucidated. This study aimed to clarify the regulatory role of σ-1R in a mouse model combining preoperative sleep deprivation with exploratory laparotomy, focusing on Keap1/Nrf2 signaling, and to evaluate its clinical translational potential. Behavioral, molecular biological, and morphological methods were utilized. Sleep deprivation exacerbated postoperative cognitive impairment, accompanied by a compensatory upregulation of σ-1R expression in hippocampal microglia. Notably, exogenous potentiation of σ-1R with the agonist PRE-084 reversed cognitive deficits, suppressed neuroinflammation and oxidative stress, promoted Nrf2 nuclear translocation, and upregulated downstream genes HO-1 and NQO1; conversely, the antagonist BD-1047 worsened the damage. Mechanistically, σ-1R physically associated with Keap1, promoting Nrf2 release. The Nrf2 inhibitor ML385 blocked PRE-084’s protective effects. In vitro experiments confirmed PRE-084 alleviated inflammation and oxidative stress via an Nrf2-dependent pathway. Moreover, σ-1R signaling may partially contribute to the neuroprotective effects of dexmedetomidine. In conclusion, this study reveals that σ-1R activation is associated with protective effects in sleep deprivation-exacerbated POCD, potentially through regulating the Keap1/Nrf2 signaling pathway, a mechanism rarely reported in this context, suggesting σ-1R as a potential avenue for further investigation in POCD prevention.

## Graphical abstract

**Figure:**
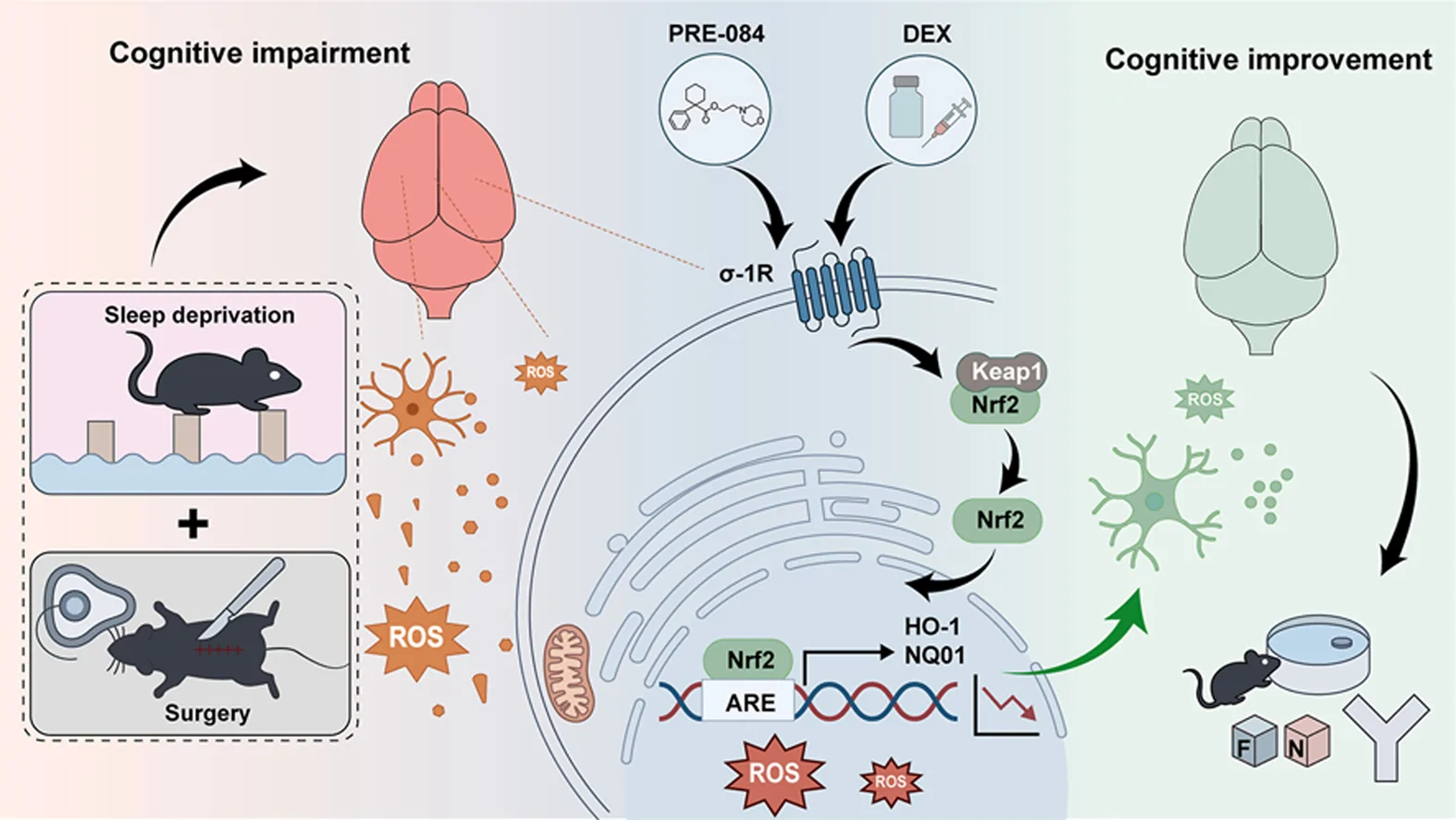

## Introduction

Postoperative cognitive dysfunction (POCD) represents a prevalent and clinically significant neurological complication characterized by persistent deficits in memory, attention, and spatial cognition [1], [2]. Beyond severely compromising patients’ quality of life and prolonging rehabilitation, POCD is increasingly recognized as a critical perioperative concern due to its potential association with the progression of long-term neurodegenerative diseases [3]. Among the myriad risk factors, perioperative sleep disturbance has emerged as a pivotal modifiable element, often precipitated by preoperative anxiety, acute pain, and environmental maladaptation [4]. Accumulating evidence indicates that sleep disruption critically lowers the brain’s tolerance threshold to surgical and anesthetic insults by disrupting cerebral redox homeostasis, activating microglia-mediated neuroinflammation, and impairing hippocampal synaptic plasticity [5]. Central to this pathological cascade is the interplay between oxidative stress driven primarily by excessive reactive oxygen species (ROS) accumulation and neuroinflammation, a vicious cycle that is now widely regarded as the core mechanism underlying the onset and progression of POCD [6], [7].

The sigma-1 receptor (σ-1R), a molecular chaperone abundantly expressed on the mitochondrial-associated endoplasmic reticulum membranes of the central nervous system, functions as an intracellular “stress sensor” that regulates diverse physiological and pathological processes, including calcium homeostasis, inflammatory responses, and oxidative stress [8]. In recent years, the neuroprotective properties of σ-1R have positioned it as a promising therapeutic target for various neurological disorders. Substantial evidence demonstrates that σ-1R activation confers robust protection across multiple disease models via the modulation of distinct cellular signaling pathways [9]. Notably, the core protective mechanism of σ-1R appears to be closely linked to the activation of the nuclear factor erythroid 2-related factor 2 (Nrf2) signaling pathway. As a pivotal antioxidant axis, the Kelch-like ECH-associated protein 1 (Keap1)/Nrf2 pathway plays a critical role in maintaining redox homeostasis and regulating anti-inflammatory responses, thereby constituting a fundamental defense against neurodegenerative damage [10]. Previous studies have shown that σ-1R exerts neuroprotection in various oxidative stress models by promoting Nrf2 nuclear translocation and upregulating the expression of downstream antioxidant genes such as heme oxygenase-1 (HO-1) and NAD(P)H quinone dehydrogenase 1 (NQO1) [11], [12]. However, evidence regarding the relationship between σ-1R and POCD remains scarce within the context of perioperative neurocognitive protection. It is currently unclear whether modulating σ-1R can mitigate the exacerbated cognitive impairment induced by preoperative sleep deprivation or how it precisely regulates the Nrf2 pathway at the molecular level. Furthermore, despite the therapeutic promise of σ-1R, a critical translational gap persists. The absence of specific clinical interventions targeting this pathway, coupled with the pharmacological limitations and safety concerns surrounding currently approved drugs, renders them unsuitable for the acute management of perioperative cognitive protection, thus representing a major bottleneck in translating basic research findings into clinical practice.

This study aimed to elucidate the protective effects and molecular mechanisms of σ-1R activation in the context of preoperative sleep deprivation-exacerbated POCD, while also exploring its clinical translational potential. To achieve this, we established a mouse model combining preoperative sleep deprivation with exploratory laparotomy and systematically evaluated the therapeutic efficacy of σ-1R modulation using in vivo pharmacological interventions and in vitro cellular assays. To further dissect the underlying molecular mechanisms, we investigated the regulatory effects of σ-1R on the Keap1/Nrf2 signaling pathway in animal models and validated the necessity of this pathway for neuroprotection using gene silencing techniques in vitro. Addressing the aforementioned translational bottleneck, we further assessed the therapeutic effects of dexmedetomidine (Dex), a sedative agent commonly used in clinical practice, on this pathological process, confirming that its neuroprotective actions are closely associated with σ-1R activation. Collectively, our findings not only reveal the feasibility of targeting the σ-1R/Keap1/Nrf2 axis but also provide novel insights and potential strategies for the clinical prevention and treatment of POCD associated with high-risk perioperative factors.

## Materials and methods

### Animals and experimental design

Male wild-type C57BL/6 J mice (aged 8–12 weeks, weighing 20–25 g) were obtained from Beijing Vitalstar Biotechnology Co., Ltd. All animals were housed under standard specific pathogen-free conditions with a 12 h light/dark cycle, a controlled temperature of 24 ± 2 °C, and relative humidity of 50%–60%, with free access to food and water. Prior to experimentation, all mice were allowed to acclimate to the laboratory environment for 7 days. The experimental protocols were approved by the Institutional Animal Care and Use Committee of the Academy of Military Medical Sciences (Approval No. DWZX-IACUC-2026-505), and all procedures complied with the institutional ethical guidelines.

Mice were randomly assigned to groups using a computer-generated random number table. All behavioral tests, tissue processing, and data analyses were performed by investigators blinded to group allocation. No animal died or was excluded during the entire study. The study consisted of four independent parts, each with its own set of groups. A total of 17 groups were included (10 mice per group, 170 mice in total). Note that the same group labels across different parts correspond to independent batches of animals and are not shared.

#### Part I: Effects of preoperative sleep deprivation on cognitive function and hippocampal σ-1R expression

Mice were randomly assigned to four groups: Control (C), sleep deprivation alone (SD), surgery alone (S), and sleep deprivation combined with surgery (SS). No pharmacological interventions were administered in this part.

#### Part II: Role of σ-1R in postoperative cognition, neuroinflammation, and oxidative stress

Mice were randomly divided into four groups: Control (C), SS, σ-1R agonist (PRE-084, Sigma-Aldrich, P2607, St. Louis, MO, USA) treatment (SS + P), and σ-1R antagonist (BD-1047, Sigma-Aldrich, B8562, St. Louis, MO, USA) treatment (SS + B). PRE-084 and BD-1047 were dissolved in normal saline and administered via intraperitoneal injection prior to surgery. Mice in the C and SS groups received an equal volume of normal saline at corresponding time points.

#### Part III: Mediation of neuroprotection by the Keap1/Nrf2 signaling pathway

Mice were randomly allocated into five groups: Control (C), SS, vehicle control (SS + V), σ-1R agonist (SS + P), and σ-1R agonist combined with Nrf2 inhibitor (ML385, Sigma-Aldrich, SML1833, St. Louis, MO, USA) treatment (SS + P + M). PRE-084 was dissolved in normal saline. ML385 was dissolved in a 5% DMSO/corn oil mixture. Mice in the SS + V group received an equal volume of the 5% DMSO/corn oil vehicle via intraperitoneal injection, matching the solvent, volume, route, and schedule of the ML385-treated groups.

#### Part IV: Effect of Dex on POCD and its association with σ-1R

Mice were randomly assigned to four groups: Control (C), SS, Dex (Sigma-Aldrich, SML0956, St. Louis, MO, USA) treatment (SS + D), and Dex combined with σ-1R antagonist treatment (SS + D + B). Both Dex and BD-1047 were prepared in normal saline. Dex was administered via tail vein injection prior to surgery. Mice in the C and SS groups received an equal volume of normal saline via intravenous injection at corresponding time points.

### Sleep deprivation

A modified multiple platform method was employed to establish the rapid eye movement (REM) sleep deprivation model in mice, following protocols described in previous studies [13]. Mice were placed in a rectangular tank measuring 41 × 21 × 20 cm, which contained eight circular platforms. Each platform had a diameter of 3 cm and protruded 6 cm above the base of the tank, with the surrounding water level maintained at 1 cm below the platform surface. No more than six mice were housed per tank. During REM sleep, the characteristic muscle atonia caused mice to lose postural tone and fall into the water, resulting in immediate awakening. The height of the platforms allowed mice to climb back onto the surface quickly, preventing drowning. The spacing between platforms was set at 5 cm, allowing animals to move freely, jump between platforms, and interact socially. This design minimized the social isolation stress caused by single housing and reduced the psychological pressure associated with forced immobility. A grid-shaped rack was fixed above the tank to hold food and water bottles, ensuring ad libitum access to food and water throughout the experiment. The water in the tanks was changed daily, and feces and food debris were removed. The water temperature was strictly maintained between 25 and 28 °C. Prior to the formal experiment, all mice were acclimated to the apparatus for 24 h without deprivation, during which they were allowed to move freely on the platforms to reduce stress induced by the novel environment.

### Anesthesia and surgery

Mice were fasted for 6–8 h prior to the operation. Anesthesia was induced using 5% isoflurane in oxygen, and induction was confirmed by the loss of the pedal withdrawal reflex and the absence of response to noxious stimuli. The mice were then secured in a supine position on a homeothermic pad maintained at 37 °C to prevent hypothermia. Anesthesia was maintained throughout the procedure via inhalation of 1–2% isoflurane delivered through a facemask. The abdominal fur was removed using depilatory cream over an area extending from the xiphoid process to the pubic symphysis and laterally to the mid-axillary lines. The surgical site was sterilized sequentially with 75% ethanol and povidone-iodine solution, followed by draping with sterile surgical towels. A 1–2 cm midline vertical incision was made through the skin and subcutaneous tissue. The linea alba and peritoneum were incised carefully, with meticulous hemostasis performed to avoid injury to visceral organs. Following entry into the peritoneal cavity, a systematic exploration of the abdominal organs, including the liver, gallbladder, stomach, intestines, spleen, and kidneys, was conducted to record their size, color, and gross morphology. After exploration, the peritoneum and linea alba were closed in one layer, followed by closure of the subcutaneous tissue and skin. The integrity of the wound was inspected immediately after suturing. For postoperative analgesia, 0.2 mL of 2% ropivacaine was injected subcutaneously around the incision margins. The mice were subsequently placed on a heating pad to recover from anesthesia. Vital signs were monitored continuously until full recovery. Prophylactic antibiotic and fluid supplementation were administered only when predefined clinical criteria were met: evidence of wound dehiscence, purulent discharge, lethargy, or body weight loss exceeding 20% of preoperative weight. No animal in this study met any of these criteria. Once awake, the mice were returned to their original cages for postoperative care.

### Behavioral assessment

#### Morris water maze (MWM)

The MWM test was employed to evaluate spatial learning and memory in rodents, following established protocols [14]. The apparatus consisted of a circular pool (diameter: 100 cm; height: 50 cm) filled with water to a depth of 30 cm and maintained at 22–25 °C. A circular escape platform (diameter: 5 cm) was submerged 1 cm below the water surface. The water was rendered opaque by the addition of non-toxic white pigment to obscure the platform. Distinct geometric patterns were affixed to the walls of each quadrant to serve as visual cues. The experiment comprised two phases. First, the spatial acquisition trial lasted for 5 consecutive days. Mice were placed into the water facing the wall at one of four starting positions. If a mouse failed to locate the platform within 60 s, it was guided to the platform and allowed to remain there for 15 s. Escape latency and swim distance were recorded. Second, the spatial probe trial was conducted 24 h after the last training session. The platform was removed, and the mouse was released from the quadrant opposite the target quadrant. The time taken to reach the former platform location, the number of crossings over the platform location, and the swim speed were recorded over a 60 s period. The percentage of time spent in the target quadrant was calculated as a measure of spatial memory retention.

#### Novel object Recognition (NOR)

The NOR test is based on the innate tendency of rodents to explore novel objects over familiar ones. It was used to assess recognition memory, following previously described methods [15]. The experiment consisted of three stages. During the habituation phase, mice were allowed to freely explore an empty square arena (50 × 50 cm) for 6 min. During the training phase, two identical objects were placed symmetrically in the arena, and mice were allowed to explore for 6 min. After training, mice were returned to their home cages for a 30-min inter-trial interval. During the test phase, one of the objects was replaced with a novel object differing in shape and color. Exploration was defined as the nose being within 2 cm of the object or touching it. Each mouse was recorded for 6 min. Between trials, the objects and the arena were thoroughly cleaned with 70% ethanol to eliminate olfactory cues. The discrimination index (Ri) was calculated as Ri = T_n_/(T_n_ + T_f_), where T_n_ is the time spent exploring the novel object and T_f_ is the time spent exploring the familiar object. A higher Ri indicates better recognition memory.

#### Y-maze

The Y-maze test was utilized to evaluate short-term spatial working memory [16]. The apparatus consisted of three arms (A, B, and C) arranged at 120° angles, each measuring 30 cm (length) × 10 cm (width) × 20 cm (height). Mice were placed in the center of the maze and allowed to explore freely for 6 min. Arm entries were recorded when all four paws entered a specific arm. A spontaneous alternation was defined as a sequence of three consecutive entries into three different arms (e.g., A→B→C). Between subjects, the maze was cleaned with 70% ethanol to remove residual odors. The spontaneous alternation percentage was calculated using the formula: [(Number of spontaneous alternations)/(Total number of arm entries − 2)] × 100%. Higher alternation rates indicate intact spatial working memory, whereas a significant reduction suggests impaired short-term memory and random exploration behavior.

### Cell culture

The murine microglial cell line BV2 (purchased from the Chinese Academy of Sciences Cell Bank) was cultured in high-glucose Dulbecco’s Modified Eagle Medium supplemented with 10% fetal bovine serum, 100 U/mL penicillin, and 100 μg/mL streptomycin. Cells were maintained in a humidified incubator at 37 °C with 5% CO_2_, and the culture medium was refreshed daily. When the cells reached 80%–90% confluence, they were detached with 0.05% trypsin and subcultured. Cells in the logarithmic growth phase were used for all subsequent experiments. To induce an inflammatory phenotype, cells were treated with lipopolysaccharide (LPS) at a concentration of 1 μg/mL for 24 h.

### Cell transfection

Small interfering RNA (siRNA) targeting Nrf2 (GenePharma, Shanghai, China) was used to silence Nrf2 expression in BV2 cells. The sequences for Nrf2-siRNA were sense: 5′-GGAAGCUGGAGAACAUUGU-3′ and antisense: 5′-ACAAAUGUUCUCCAGCUUCC-3′. A negative control siRNA (NC-siRNA) with the sequence sense: 5′-UUCUUCGAACGUGUCACGU-3′ and antisense: 5′-ACGUGACACGUUCGGAGAA-3′ was used as the control. BV2 cells were transfected with either Nrf2-siRNA or NC-siRNA using a standard transfection reagent. The silencing efficiency was confirmed by RT-qPCR and WB analysis 48 h post-transfection.

### Cell viability assay

BV2 cells in the logarithmic growth phase were seeded into 96-well plates at a density of 5 × 10^3^ cells per well in 200 μL of medium. After 24 h of incubation to allow adhesion, cells were subjected to the designated treatments. Subsequently, cells were subjected to the designated pharmacological treatments and stimulated with lipopolysaccharide (LPS, *Escherichia coli*serotype O111:B4, MedChemExpress, HY-D1056, Monmouth Junction, NJ, USA) at 1 μg/mL. Each experimental group was set up with six replicates. Following the intervention, 10 μL of Cell Counting Kit-8 (CCK-8) reagent was added to each well, and the plates were incubated for an additional 2 h at 37 °C in a 5% CO_2_ humidified atmosphere. The optical density (OD) was measured at a wavelength of 450 nm using a microplate reader. Cell viability was expressed as a percentage relative to the control group, calculated using the formula: (OD of experimental group/OD of control group) × 100%.

### Immunofluorescence (IF)

#### Tissue immunofluorescence

Mice from each group were anesthetized and transcardially perfused with ice-cold phosphate-buffered saline (PBS) followed by 4% paraformaldehyde (PFA). Brains were rapidly harvested and post-fixed in 4% PFA for 24 h at 4 °C. Subsequently, tissues were dehydrated in a graded sucrose series until they sank. The brains were then embedded in optimal cutting temperature (OCT) compound, and serial coronal frozen sections (5 μm thickness) were prepared. Sections were air-dried at room temperature, washed three times with PBS (5 min per wash), permeabilized with 0.3% Triton X-100 for 20 min, and blocked with 5% bovine serum albumin (BSA) for 1 h at room temperature.

Sections were incubated overnight at 4 °C with the following primary antibodies diluted in blocking buffer: anti-Sigma-1 receptor (Santa Cruz, sc-137075, Dallas, TX, USA; 1:200), anti-GFAP (Proteintech, 16825-1-AP, Wuhan, China; 1:500), anti-NeuN (Proteintech, 26975-1-AP, Wuhan, China; 1:200), and anti-Iba1 (Proteintech, 10904-1-AP, Wuhan, China; 1:500). After returning to room temperature for 30 min and washing with PBS, sections were incubated with an Alexa Fluor® 488-conjugated goat anti-rabbit IgG secondary antibody (Invitrogen, A-11034, Carlsbad, CA, USA; 1:500) and Alexa Fluor® 594-conjugated goat anti-mouse IgG (Invitrogen, A-11032, Carlsbad, CA, USA; 1:500) for 1 h at room temperature in the dark. Nuclei were counterstained with 4′,6-diamidino-2-phenylindole (DAPI) for 5 min, mounted with antifade mounting medium, and visualized under a fluorescence microscope. Fluorescence intensity was quantitatively analyzed using ImageJ software (Version 10.8.1, BD Life Sciences, USA).

#### Cellular immunofluorescence (ICC)

For immunocytochemistry, BV2 cells were seeded onto coverslips in 12-well plates at a density of 1 × 10^4^ cells per well. Following the designated pharmacological treatments and LPS induction, cells were washed three times with PBS. Cells were fixed with 4% PFA for 15 min at room temperature, permeabilized with 0.3% Triton X-100 for 10 min, and blocked with 5% BSA for 30 min. Cells were then incubated overnight at 4 °C with primary antibodies against σ-1R and Nrf2 (Cell Signaling Technology, CST-12721, Danvers, MA, USA). After rewarming and washing, cells were incubated with fluorophore-conjugated secondary antibodies for 50 min at room temperature in the dark. Nuclei were stained with DAPI for 5 min, and coverslips were mounted onto glass slides using antifade mounting medium. Images were acquired using a fluorescence microscope.

### Western blotting (WB)

#### Tissue protein extraction and cytoplasmic/nuclear fractionation

Hippocampal tissues were harvested from mice in each group. For cytoplasmic protein extraction, tissues were homogenized in cytoplasmic extraction buffer (containing protease and phosphatase inhibitors) at a weight-to-volume ratio of 1:10. The homogenate was centrifuged at 10,000 rpm for 10 min at 4 °C, and the supernatant was collected as the cytoplasmic protein fraction. For nuclear protein extraction, the remaining pellet was resuspended in nuclear extraction buffer and lysed by vortexing on ice for 30 min, with vortexing every 10 min. The lysate was centrifuged at 12,000 rpm for 30 min at 4 °C, and the resulting supernatant was collected as the nuclear protein fraction.

#### Protein electrophoresis and transfer

Protein concentrations were determined using a Bicinchoninic acid (BCA) assay kit, and protein concentrations were normalized across all samples. Samples were mixed with 5 × loading buffer and denatured by boiling at 95 °C for 10 min. Equal amounts of protein were separated by 10%–12% SDS-PAGE gels and subsequently transferred onto polyvinylidene fluoride membranes.

#### Immunoblotting and quantification

Membranes were blocked with 5% non-fat dry milk at room temperature for 1 h and incubated overnight at 4 °C with the following primary antibodies: anti-IL-1β (Abcam, ab283818, Cambridge, UK; 1:1000), anti-IL-6 (Abcam, ab259341, Cambridge, UK; 1:1000), anti-TNF-α (Abcam, ab183218, Cambridge, UK; 1:1000), anti-IL-4 (Bioss, bs-0581 R, Beijing, China; 1:500), anti-IL-10 (Abcam, ab189392, Cambridge, UK; 1:1000), anti-Sigma-1 receptor (Proteintech, 15168-1-AP, Wuhan, China; 1:1000), anti-Nrf2 (Cell Signaling Technology, 12721 T, Danvers, MA, USA; 1:1000), anti-Keap1 (Proteintech, 10503-2-AP, Wuhan, China; 1:1000), anti–HO–1 (Proteintech, 10701-1-AP, Wuhan, China; 1:1000), anti-NQO1 (Proteintech, 11451-1-AP, Wuhan, China; 1:1000), anti-β-actin (Proteintech, 66009-1-Ig, Wuhan, China; 1:5000), and anti-Histone H3 (Proteintech, 17168-1-AP, Wuhan, China; 1:1000).

After three washes with TBST (10 min per wash), membranes were incubated with a horseradish peroxidase-conjugated goat anti-rabbit IgG secondary antibody (Jackson ImmunoResearch, 111-035-003, West Grove, PA, USA; 1:5000) for 1 h at room temperature. Protein bands were visualized using an enhanced chemiluminescence substrate and imaged. The grayscale intensity of the bands was quantified using ImageJ software. β-actin served as the loading control for cytoplasmic proteins, while Histone H3 served as the loading control for nuclear proteins. Relative protein expression levels were calculated accordingly.

#### Cellular protein extraction

BV2 cells were washed three times with PBS after the designated treatments. Total cellular proteins were extracted using radioimmunoprecipitation assay buffer lysis buffer (containing protease and phosphatase inhibitors) on ice for 30 min. Cells were scraped, collected into centrifuge tubes, and centrifuged at 12,000 rpm for 30 min at 4 °C. The supernatants were collected as total protein extracts. Subsequent procedures, including protein quantification, electrophoresis, and immunoblotting, were performed identically to those described for the tissue samples.

### Co-immunoprecipitation (Co-IP)

Mouse hippocampal tissues were harvested and rinsed with ice-cold PBS, followed by mincing into small pieces. Tissues were homogenized in IP lysis buffer containing protease inhibitors and incubated on ice. The lysates were centrifuged at 4 °C, and the supernatants were collected. Protein concentrations were determined using a BCA assay kit. To prepare the beads, Protein A/G beads were washed with PBS and resuspended as a 50% slurry. To preclear the lysates and remove nonspecific binding, 1 mL of total protein lysate was incubated with 1 μg of control isotype IgG and 100 μL of the bead slurry for 1 h at room temperature with rotation. The samples were centrifuged, and the precleared supernatants were collected. For the immunoprecipitation reaction, the precleared supernatants were incubated with an anti-Keap1 primary antibody (1 μg per 1 mg of total protein) and 100 μL of the bead slurry overnight at 4 °C with rotation. An isotype IgG control was included as a negative control. Following incubation, the immunocomplexes were pelleted by centrifugation, washed three times with ice-cold PBS, and resuspended in 1 × loading buffer. The samples were boiled at 100 °C for 5 min to elute and denature the proteins. The eluted samples, along with an input control, were subsequently analyzed by WB as described above. The membranes were probed with anti-Sigma-1 receptor and anti-Nrf2 antibodies to detect the presence of these proteins within the Keap1 immunoprecipitation complex.

### Reverse transcription quantitative polymerase chain reaction (RT-qPCR)

Total RNA was extracted from mouse hippocampal tissues using TRIzol reagent (Invitrogen, Carlsbad, CA, USA) according to the manufacturer’s protocol. RNA concentration and purity were determined using a UV spectrophotometer. One microgram of total RNA was reverse transcribed into complementary DNA (cDNA) using a reverse transcription kit (TaKaRa, Shiga, Japan), following the manufacturer’s instructions. The reaction conditions were 42 °C for 60 min, followed by 70 °C for 15 min to terminate the reaction. Quantitative real-time PCR (qPCR) was performed using a SYBR Green-based detection system. The 20 μL reaction mixture consisted of SYBR Green master mix, forward and reverse primers, cDNA template, and nuclease-free water. The amplification protocol included an initial denaturation at 95 °C for 3 min, followed by 40 cycles of denaturation at 95 °C for 10 s and annealing/extension at 60 °C for 30 s. Melting curve analysis was conducted to verify the specificity of the amplified products. Primer sequences were designed based on the GenBank database and are listed in Table 1. The relative expression levels of target genes were calculated using the 2^−^ΔΔCt method, with GAPDH serving as the internal reference gene. For BV2 cells, following the designated treatments, the culture medium was discarded, and cells were washed three times with PBS. Total RNA was extracted directly using TRIzol reagent, and subsequent reverse transcription and qPCR procedures were performed identically to those described for the tissue samples.

**Table 1:** Primer sequences used for RT-qPCR.

Target	Sequences (5′–3′)	
	Forward	Reverse
Sigma-1	TGGATGGGCGCCATGT	AGGGCGGTGCCGAAGAG
IL-1β	CACTACAGGCTCAGAGATGAACAAC	TGTCGTTGCTTGGTTCTCCTTGTAC
IL-6	CTTCTTGGGACTGATGCTGGTGAC	TCTGTTGGGAGTGGTATCCTCTGTG
TNF-α	ATGTCTCAGCCTCTTCTCATTC	GCTTGTCACTCGAATTTTGAGA
IL-4	TACCAGGAGCCATATCCACGGATG	TGTGGTGTTCTTCGTTGCTGTGAG
IL-10	AGAGAAGCATGGCCCAGAAATCAAG	CTTCACCTGCTCCACTGCCTTG
Keap1	GATATGAGCCAGAGCGGGAC	CATACAGCAAGCGGTTGAGC
Nrf2	CTAGCTAGCTAGAATGCGATA	CTGCAAGCATGCGAGATAGCTAA
HO-1	TTGAGGAGCTGCAGGTGATG	TGTCTGGGATGAGCTAGTGC
NQO1	GTCTGGAAACCGTCTGGGAG	GAATGGACTTGCCCAGGTGA
GAPDH	CGATCTTATTAGCAGAGATCG	TGTGAACGGATTTGGCCGTA

### Measurement of oxidative stress markers

For the assessment of oxidative stress markers in hippocampal tissues, tissues were homogenized in pre-chilled normal saline at a weight-to-volume ratio of 1:10. The homogenates were centrifuged at 10,000 rpm for 15 min at 4 °C, and the supernatants were collected for analysis. For cellular assays, BV2 cells were washed three times with PBS following the designated treatments. Cells were lysed with pre-chilled lysis buffer on ice for 30 min, followed by centrifugation at 12,000 rpm for 15 min at 4 °C. The resulting supernatants were collected for subsequent measurements.

The levels of malondialdehyde (MDA) and glutathione (GSH), as well as the activities of glutathione peroxidase (GPX) and superoxide dismutase (SOD), were quantified using commercially available assay kits (Beyotime Biotechnology, Shanghai, China) according to the manufacturer’s instructions. Specifically, MDA levels were determined using the thiobarbituric acid method by measuring the absorbance at 532 nm. GSH content was measured using the dithionitrobenzoic acid method at 412 nm. GPX activity was assessed using a hydrogen peroxide substrate method by monitoring the change in absorbance at 412 nm. SOD activity was evaluated using a xanthine oxidase method by measuring the absorbance at 550 nm. All samples were analyzed in triplicate to ensure reliability. The final concentrations and activities were calculated based on the standard curves and formulas provided by the respective kits.

### Reactive oxygen species (ROS) assay

Intracellular ROS levels in BV2 cells were measured using a ROS assay kit (Beyotime Biotechnology, Shanghai, China) based on the fluorescent probe 2′,7′-dichlorodihydrofluorescein diacetate (DCFH-DA). Following the designated pharmacological treatments, the culture medium was removed, and cells were washed twice with PBS. The cells were then incubated with DCFH-DA diluted in serum-free medium to a final concentration of 10 μM at 37 °C in a 5% CO_2_ incubator for 20 min in the dark. After incubation, cells were washed three times with PBS to remove any unloaded probe.

ROS production was assessed using two complementary methods:

Confocal Microscopy: For cells seeded on coverslips, intracellular ROS-specific fluorescence was directly observed using a laser scanning confocal microscope. The excitation and emission wavelengths were set at 488 nm and 525 nm, respectively. Images were acquired, and the fluorescence intensity was quantitatively analyzed using ImageJ software.

Flow Cytometry: Adherent cells were detached using 0.25% trypsin, collected, and resuspended in PBS to generate a single-cell suspension. The fluorescence intensity of DCF was detected using a flow cytometer with excitation at 488 nm and emission at 525 nm. A total of 1 × 10^4^ events were recorded for each sample. The data were analyzed using FlowJo software (Version 10, BD Life Sciences, USA) to determine the proportion of ROS-positive cells and the mean fluorescence intensity.

All experiments were performed in triplicate to ensure reliability.

### Modeling and simulation of physiologically based pharmacokinetics (PBPK)

A whole-body PBPK model of DEX was developed using PK-Sim® software (Version 11.0.250, Bayer AG, Germany). The “AI-PBPK” module of the software was utilized. The SMILES code of DEX (C[C@@H](C1=CC=CC(C)=C1C)C2=CNC=N2) and standard human parameters (body weight of 60 kg) were input. Following this, the automated prediction and calculation function of the software was executed to generate and obtain the distribution volume parameters (log Kp) for each tissue.

### Molecular docking

Molecular docking was performed as follows: First, the crystal structure of the σ-1 receptor (σ-1R; UniProt ID: Q99720) was retrieved and downloaded from the UniProt database. Based on the reported active site information of this crystal structure, the three-dimensional structure of the ligand DEX was obtained from the PubChem database and saved in SDF format. The receptor protein was processed using PyMOL (Version 2.5.0, Schrödinger, LLC) to remove water molecules and the original ligand. Hydrogen addition and charge assignment were then carried out using AutoDock Tools (Version 1.5.7, The Scripps Research Institute). Subsequently, both the receptor and ligand were converted to the PDBQT format. The molecular docking simulation was executed using AutoDock Vina (Version 1.1.2, The Scripps Research Institute). Analysis of the binding modes was conducted with the PLIP tool (Version 2.3.0, Leibniz Institute of Molecular Pharmacology). The optimal conformation was selected based on the calculated binding free energy as the evaluation metric, and the results were visualized using PyMOL.

### Statistical analysis

All data were statistically analyzed using GraphPad Prism software (Version 10.1.2, GraphPad Software, USA) and are presented as the mean ± standard deviation(SD). Comparisons between two independent groups were performed using an unpaired two-tailed Student’s *t*-test. For comparisons among multiple groups, one-way or two-way analysis of variance (ANOVA) was applied, followed by Tukey’s test for post hoc pairwise comparisons. The Pearson correlation coefficient was used to evaluate linear relationships. For all statistical analyses, a significance level (α) of 0.05 was applied, and a two-tailed *P* < 0.05 was considered statistically significant.

## Results

### Preoperative sleep deprivation exacerbates surgery-induced cognitive impairment in mice

We first evaluated the effects of different durations of sleep deprivation (24, 48, and 72 h) on cognitive and motor functions in mice. To assess the physiological responses, we measured body weight and serum corticosterone concentrations, which showed a time-dependent reduction in body weight and altered corticosterone levels across groups (Fig. S1A–B). Notably, 24 h of sleep deprivation did not induce significant cognitive deficits or alterations in locomotor activity (Fig. S1C-L). Therefore, this duration was selected for subsequent experiments to establish a sleep deprivation model without baseline cognitive impairment, allowing specific investigation of its exacerbating effect on surgery-induced cognitive deficits.

Mice subjected to 24 h of sleep deprivation underwent laparotomy. Their learning and memory abilities were then systematically assessed using the MWM, NOR, and Y-maze tests to determine the impact of preoperative sleep deprivation on postoperative cognitive function. In the MWM acquisition trials, the escape latency and path length to the platform decreased over training days in all groups (Fig. 1B–D), indicating no significant difference in basic learning ability. During the spatial probe trial, mice in the surgery-only group (S group) exhibited only a significant reduction in the percentage of time spent in the target quadrant (Fig. 1G), while the time to first platform crossing (Fig. 1F) and the number of platform crossings (Fig. 1H) showed a non-significant worsening trend. In contrast, the preoperative sleep deprivation plus surgery group (SS group) displayed more severe cognitive impairment, with these metrics significantly worse than those in the S group (Fig. 1F–H). The NOR test further supported this trend, as the SS group spent significantly less time exploring the novel object compared to the control (C) and S groups (Fig. 1J–K). Additionally, Y-maze results showed that the spontaneous alternation rate was already significantly lower in the S group than in the C group and was further impaired in the SS group compared to the S group (Fig. 1M-N). Importantly, there were no significant differences among groups in the average swimming speed in the MWM (Fig. 1I), the total distance moved in the NOR test (Fig. 1L), or the total number of arm entries in the Y-maze (Fig. 1O), ruling out confounding effects of motor function or exploratory motivation on the cognitive assessments. These results consistently indicate that surgical trauma induced a certain degree of cognitive impairment in mice, and preoperative sleep deprivation further exacerbated this pathological process.

**Fig. 1:**
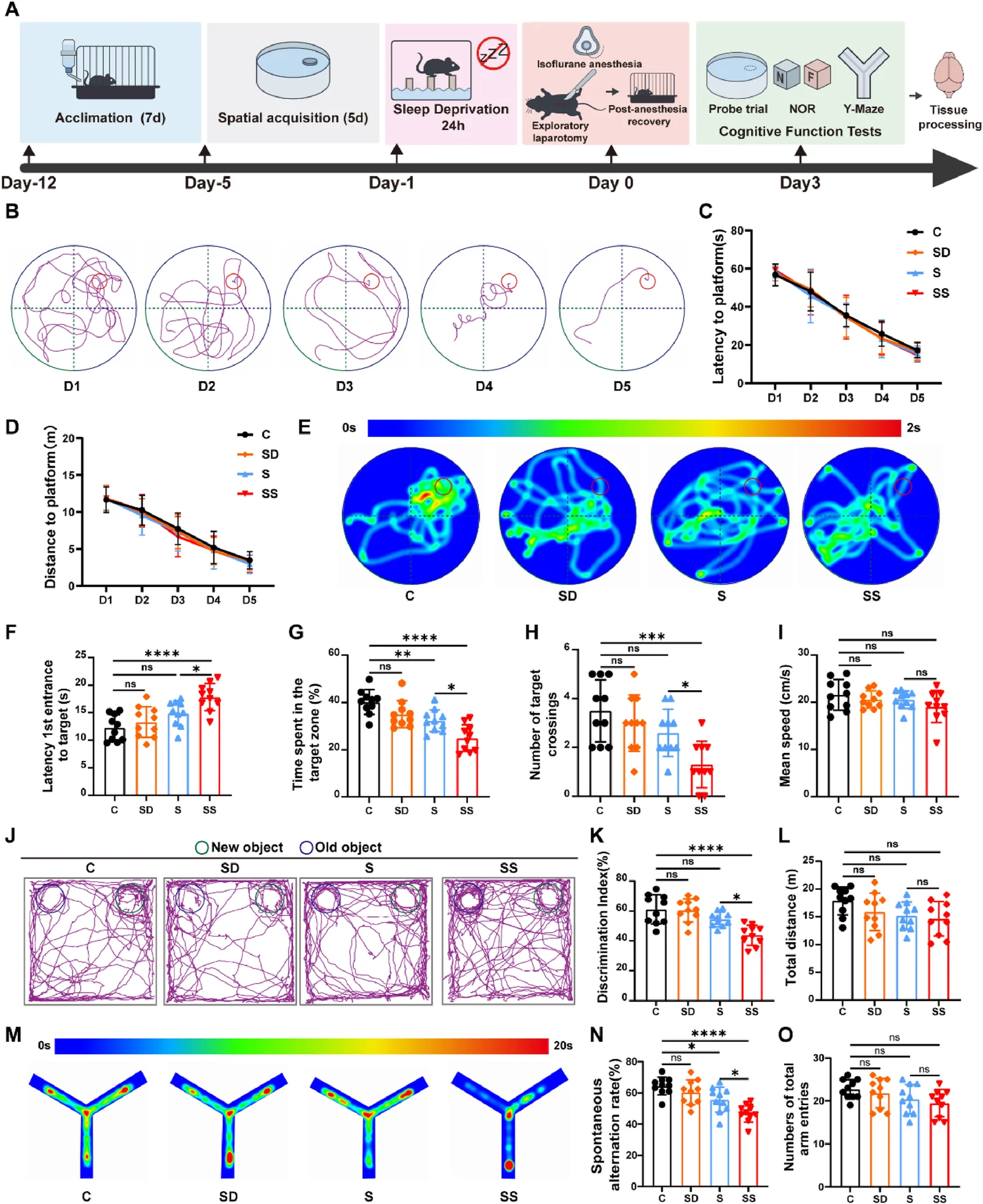
**Preoperative sleep deprivation exacerbates surgery-induced cognitive impairment in mice.** (A) Schematic of the experimental timeline. Mice were acclimated for 7 days and pre-trained in the MWM for 5 days to establish a cognitive baseline. They were then randomly assigned to four groups: Control, 24 h Sleep Deprivation (SD) alone, Surgery alone (laparotomy under isoflurane anesthesia), or Sleep Deprivation combined with Surgery (SS). Cognitive function was assessed on postoperative day 3 using the MWM spatial probe test, NOR test, and Y-maze test, followed by brain tissue collection. (B) Representative swimming paths during MWM acquisition. (C) Escape latency. (D) Path length to the platform. (E) Representative paths during the MWM probe test. (F) Time to first platform crossing. (G) Percentage of time in the target quadrant. (H) Number of platform crossings. (I) Average swimming speed. (J) Representative movement tracks in the NOR test. (K) Discrimination index. (L) Total distance traveled in NOR. (M) Representative tracks in the Y-maze. (N) Spontaneous alternation rate. (O) Total arm entries. Data are presented as mean ± SD (n = 10). Statistical significance was determined by one- or two-way ANOVA with Tukey’s post hoc test. ^ns^*P* > 0.05, ∗*P* < 0.05, ∗∗*P* < 0.01, ∗∗∗*P* < 0.001, ∗∗∗∗*P* < 0.0001.

### Preoperative sleep deprivation upregulates hippocampal σ-1R expression and alters its cellular distribution in mice after surgery

To clarify the cell-type specificity of σ-1R expression in the cognitive impairment model, we analyzed the expression and localization of σ-1R in neurons (NeuN), microglia (Iba-1) and astrocytes (GFAP) by IF co-localization. The up-regulation of σ-1R showed brain-region specificity: no significant inter-group differences in σ-1R expression were observed in the prefrontal cortex (Fig. S2A–D), whereas evident pathological changes were detected in the hippocampal Cornu Ammonis 1 (CA1) region among the groups (Fig. 2A–C, E). In hippocampal tissue of control mice, σ-1R was primarily localized in neurons, followed by astrocytes, with the lowest expression in microglia. However, in mice that developed cognitive impairment after either surgery alone (S group) or sleep deprivation combined with surgery (SS group), the fluorescence intensity of σ-1R was significantly increased in neurons, astrocytes and microglia (Fig. 2A–C). Quantitative co-localization analysis further revealed (Fig. 2D) that although σ-1R was highly co-localized with NeuN in all groups, its co-localization with Iba-1 increased most prominently in the cognitively impaired groups, suggesting that microglia may be the main cellular carriers of the upregulated σ-1R response during the pathological process of cognitive dysfunction. WB and RT-qPCR results confirmed the altered σ-1R expression: compared with the control (C) group, both protein and mRNA levels of σ-1R in the hippocampus were significantly up-regulated in the S and SS groups, with a greater increase in the SS group than in the S group (Fig. 2F–H). In summary, the cognitive impairment-related up-regulation of σ-1R was mainly confined to the hippocampal CA1 region. Notably, the induced up-regulation of σ-1R in microglia may play a key regulatory role in the process by which sleep deprivation exacerbates postoperative cognitive decline.

**Fig. 2:**
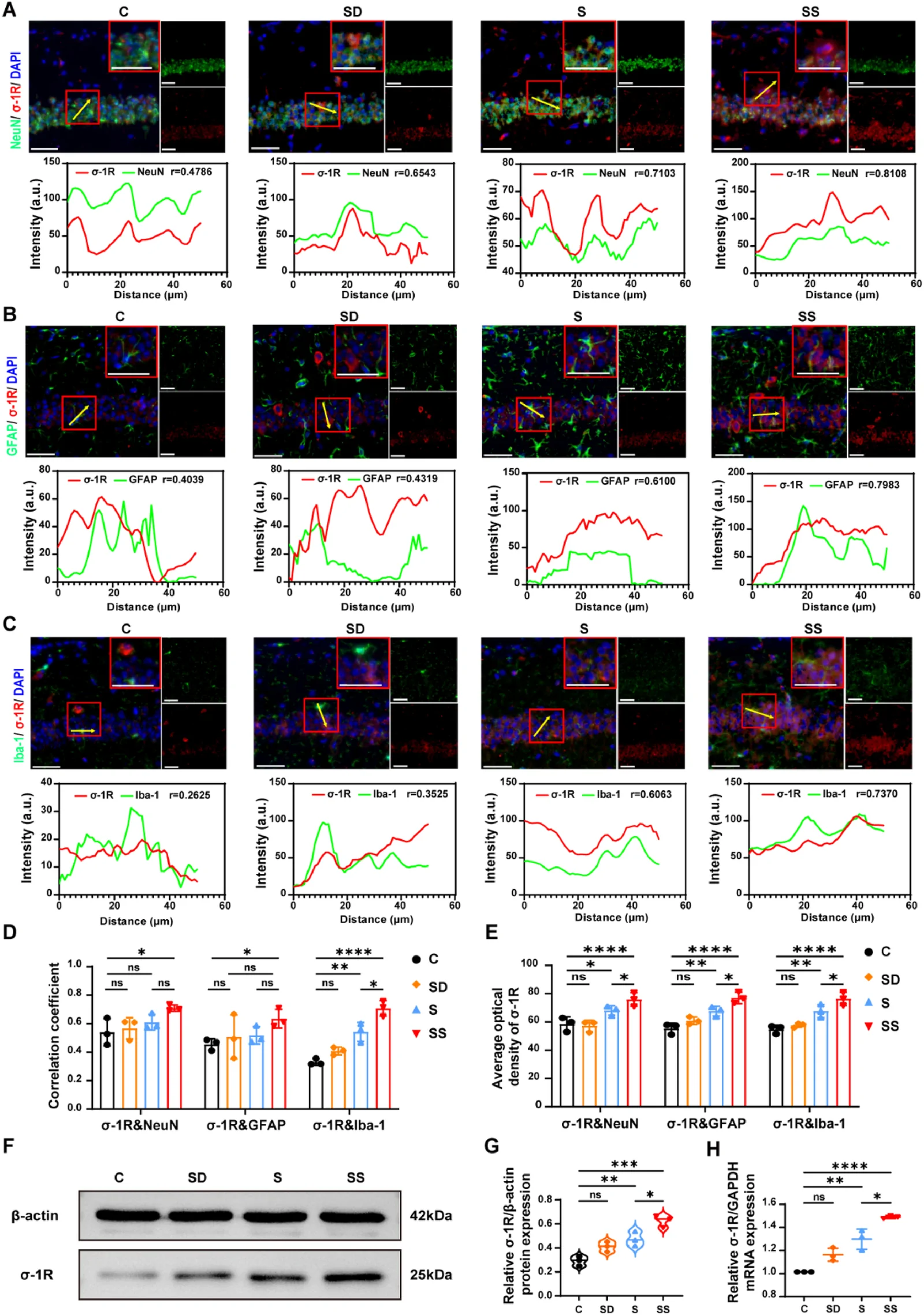
**Expression and localization of σ-1R in the mouse hippocampus.** (A–C) IF staining images of mouse brain sections. Blue: nuclei (DAPI staining). Red: σ-1R. Green: neuronal marker NeuN (A), astrocytic marker GFAP (B), and microglial marker Iba-1 (C). Higher-magnification views of the areas outlined by red boxes are shown in the upper right corners. The line graphs below display fluorescence intensity profiles along the yellow measurement lines; the Pearson correlation coefficient (r) indicates the degree of spatial co-localization between the two signals. Scale bar = 50 μm. (D) Quantitative analysis of the co-localization coefficients between σ-1R and NeuN, GFAP, or Iba-1 (n = 3 mice per group; 2 randomly selected non-overlapping fields per mouse were averaged to yield one value per mouse; the per-mouse averages were used for statistical analysis). (E) Quantification of the mean fluorescence intensity for σ-1R co-localized with NeuN, GFAP, or Iba-1 (same statistical parameters as in D). (F) Representative WB bands of σ-1R. (G) Quantitative analysis of relative σ-1R protein expression (normalized to β-actin). (H) Quantitative analysis of relative σ-1R mRNA expression (normalized to GAPDH). Data are presented as the mean ± SD; n = 3 (biological replicates). Statistical significance was determined by one-way ANOVA with Tukey’s post hoc test. ^ns^*P* > 0.05, ∗*P* < 0.05, ∗∗*P* < 0.01, ∗∗∗*P* < 0.001, ∗∗∗∗*P* < 0.0001.

### σ-1R activation effectively improves postoperative cognitive function in sleep-deprived mice

Based on the above findings, we further explored the regulatory effects of activating or inhibiting σ-1R on postoperative cognitive function in sleep-deprived mice. In the MWM acquisition trials, all groups showed similar learning curves (Fig. 3B–D). During the spatial probe test, the σ-1R agonist PRE-084 significantly attenuated cognitive impairment: mice in the SS + P group exhibited a significantly shorter latency to first cross the former platform location compared with the SS group (Fig. 3F), along with a significant increase in the percentage of time spent in the target quadrant (Fig. 3G) and the number of platform crossings (Fig. 3H). Conversely, the σ-1R antagonist BD-1047 further aggravated cognitive deficits, showing the opposite trends in these indices. No statistical difference in average swimming speed was observed among the groups (Fig. 3I), ruling out confounding effects of locomotor activity. In the NOR test, under the condition of comparable exploratory activity across the four groups (Fig. 3L), PRE-084 significantly increased the time spent exploring the novel object, whereas BD-1047 reduced it（Fig. 3J,K）. The Y-maze results corroborated these observations: the spontaneous alternation rate was significantly higher in the SS + P group than in the SS group, and it was further decreased in the SS + B group (Fig. 3M,N). Together, these data indicate that pharmacological activation of σ-1R with PRE-084 can effectively rescue the postoperative cognitive impairment exacerbated by preoperative sleep deprivation, whereas antagonism with BD-1047 worsens the deficits, suggesting that σ-1R represents a potential target for further investigation in preventing and mitigating this form of cognitive dysfunction.

**Fig. 3:**
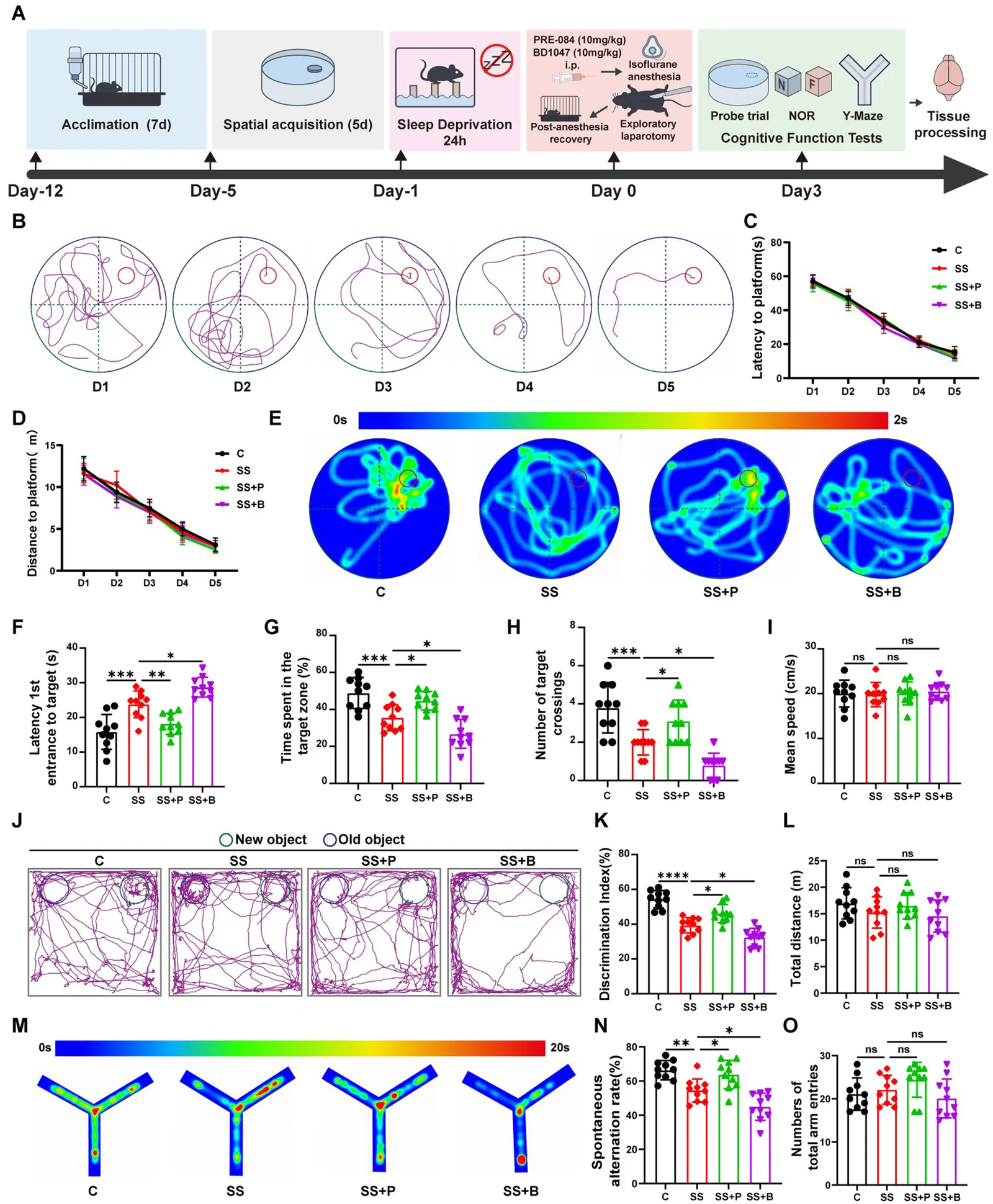
**Effect of modulating σ-1R on postoperative cognitive function in sleep-deprived mice.** (A) Schematic of the experimental timeline. Mice were acclimated for 7 days and pre-trained in the MWM for 5 days. After 24 h of preoperative sleep deprivation, mice received intraperitoneal injection of the σ-1R agonist PRE-084 (10 mg/kg) or antagonist BD-1047 (10 mg/kg) 1 h before surgery. Laparotomy was performed under isoflurane anesthesia. Cognitive function was assessed on postoperative day 3 using the MWM spatial probe test, NOR test, and Y-maze test, followed by brain tissue collection. (B) Representative swimming paths during MWM acquisition. (C) Escape latency. (D) Path length to the platform. (E) Representative paths during the MWM probe test. (F) Time to first platform crossing. (G) Percentage of time in the target quadrant. (H) Number of platform crossings. (I) Average swimming speed. (J) Representative movement tracks in the NOR test. (K) Discrimination index. (L) Total distance traveled in NOR. (M) Representative tracks in the Y-maze. (N) Spontaneous alternation rate. (O) Total arm entries. Data are presented as mean ± SD (n = 10). Statistical significance was determined by one- or two-way ANOVA with Tukey’s post hoc test. ^ns^*P* > 0.05, ∗*P* < 0.05, ∗∗*P* < 0.01, ∗∗∗*P* < 0.001, ∗∗∗∗*P* < 0.0001.

### σ-1R activation alleviates hippocampal neuroinflammation and oxidative stress and up-regulates the Nrf2 signaling pathway

To investigate whether σ-1R influences cognitive function by modulating neuroinflammation and oxidative stress, we examined relevant molecular markers. In terms of inflammatory regulation (Fig. 4A–C), WB and RT-qPCR results showed that, compared with the control (C) group, protein and mRNA levels of the pro-inflammatory cytokines IL-1β, IL-6 and TNF-α were significantly up-regulated in the hippocampus of SS group mice, while the anti-inflammatory cytokines IL-4 and IL-10 showed a compensatory upward trend, indicating an imbalance in inflammatory homeostasis. PRE-084 intervention significantly reduced the levels of pro-inflammatory factors and further enhanced the expression of anti-inflammatory factors, whereas the antagonist exacerbated this imbalance. Regarding oxidative stress (Fig. 4D–G), the activities of antioxidant enzymes (SOD, GPX) and the content of GSH were decreased, while the level of the lipid peroxidation product MDA was increased in the hippocampus of SS group mice. Agonist treatment markedly restored SOD and GPX activities and GSH content, and reduced MDA accumulation; the inhibitor further aggravated oxidative damage.

**Fig. 4:**
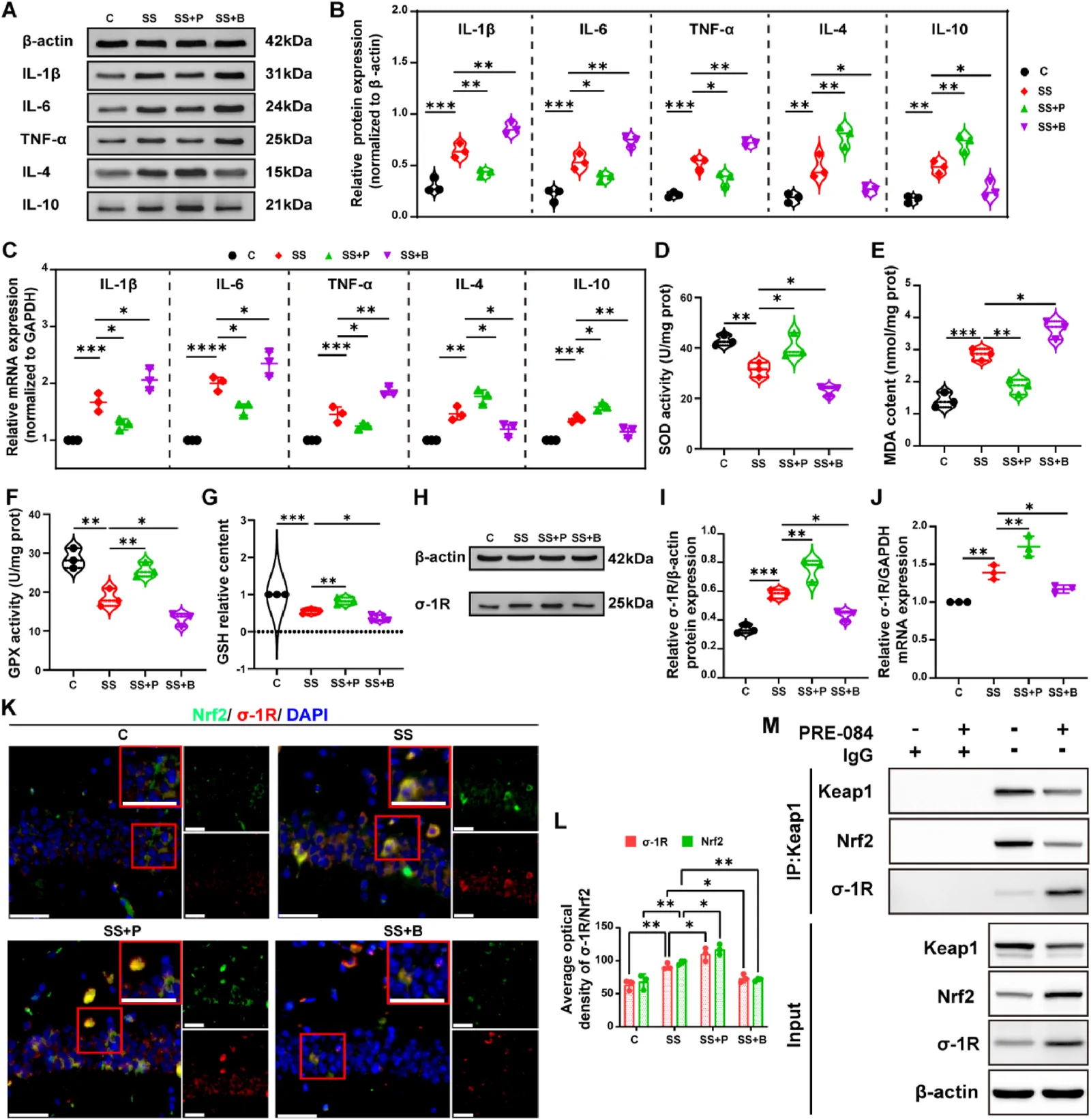
**σ-1R activation alleviates hippocampal neuroinflammation and oxidative stress and up-regulates the Nrf2 signaling pathway.** (A) Representative WB images of the inflammatory cytokines IL-1β, IL-6, TNF-α, IL-4, and IL-10 in hippocampal tissues from each group. (B) Quantitative analysis of the relative protein expression levels of the inflammatory cytokines shown in (A) (normalized to β-actin). (C) Quantitative analysis of the relative mRNA expression levels of the inflammatory cytokines (normalized to GAPDH). (D) SOD activity in hippocampal tissues. (E) MDA content in hippocampal tissues. (F) GPX activity in hippocampal tissues. (G) Relative GSH content in hippocampal tissues. (H) Representative Western blot images of σ-1R in hippocampal tissues. (I) Quantitative analysis of σ-1R protein expression (normalized to β-actin). (J) Quantitative analysis of σ-1R mRNA expression (normalized to GAPDH). (K) IF staining images of mouse brain sections. Blue: nuclei (DAPI staining). Red: σ-1R. Green: Nrf2. Higher-magnification views of the areas outlined by red boxes are shown in the upper right corners. Scale bar = 50 μm. (L) Quantitative analysis of the mean fluorescence intensity of σ-1R and Nrf2. (M) Co-IP assay analyzing the protein complex association among σ-1R, Keap1, and Nrf2. IgG served as the negative control; Input represents total protein lysate. Data are presented as the mean ± SD; n = 3 (biological replicates). Statistical significance was determined by one-way ANOVA with Tukey’s post hoc test. ^ns^*P* > 0.05, ∗*P* < 0.05, ∗∗*P* < 0.01, ∗∗∗*P* < 0.001, ∗∗∗∗*P* < 0.0001.

To explore the underlying signaling pathway, we examined the interaction between σ-1R and Nrf2. IF co-localization revealed extensive co-existence of σ-1R and Nrf2 in hippocampal tissue, and agonist treatment promoted the nuclear translocation of Nrf2 (Fig. 4K). Quantitative analysis indicated that the expression of both σ-1R and Nrf2 was up-regulated in the SS group, and agonist treatment further synergistically enhanced their expression, whereas the antagonist significantly attenuated this up-regulation (Fig. 4L). Co-immunoprecipitation (Co-IP) results showed that under normal conditions, Nrf2 co-precipitated with Keap1. After agonist treatment, σ-1R co-precipitated with Keap1, accompanied by reduced Nrf2 signal in the Keap1 immunoprecipitate. (Fig. 4M).

Taken together, these results indicate that σ-1R participates in regulating postoperative hippocampal inflammatory responses and oxidative stress. Its activation significantly suppresses neuroinflammation and alleviates oxidative stress. The observed association between σ-1R and the Nrf2 pathway suggests that its protective effects likely involve modulation of this signaling axis.

### The neuroprotective effect of σ-1R agonist is mediated via the Keap1/Nrf2 pathway through anti-inflammatory and anti-oxidative actions

To validate whether the neuroprotective effect of σ-1R depends on the Keap1/Nrf2 pathway, we combined the σ-1R agonist PRE-084 with the Nrf2 inhibitor ML385 and performed behavioral and biochemical assessments. In the MWM test, compared with the SS + P group, the SS + P + M group showed a significantly prolonged latency to first cross the platform, along with reduced time spent in the target quadrant and fewer platform crossings (Fig. 5B–F and Fig. S3A and B). The NOR test further confirmed that ML385 significantly shortened the exploration time for the novel object in the SS + P group (Fig. 5H and Fig. S3C). A similar trend was observed in the Y-maze, where ML385 reduced the spontaneous alternation rate in the SS + P group (Fig. 5J and Fig. S3D). Additionally, ML385 did not affect motor function or exploratory activity in mice (Fig. 5J, I, K).

**Fig. 5:**
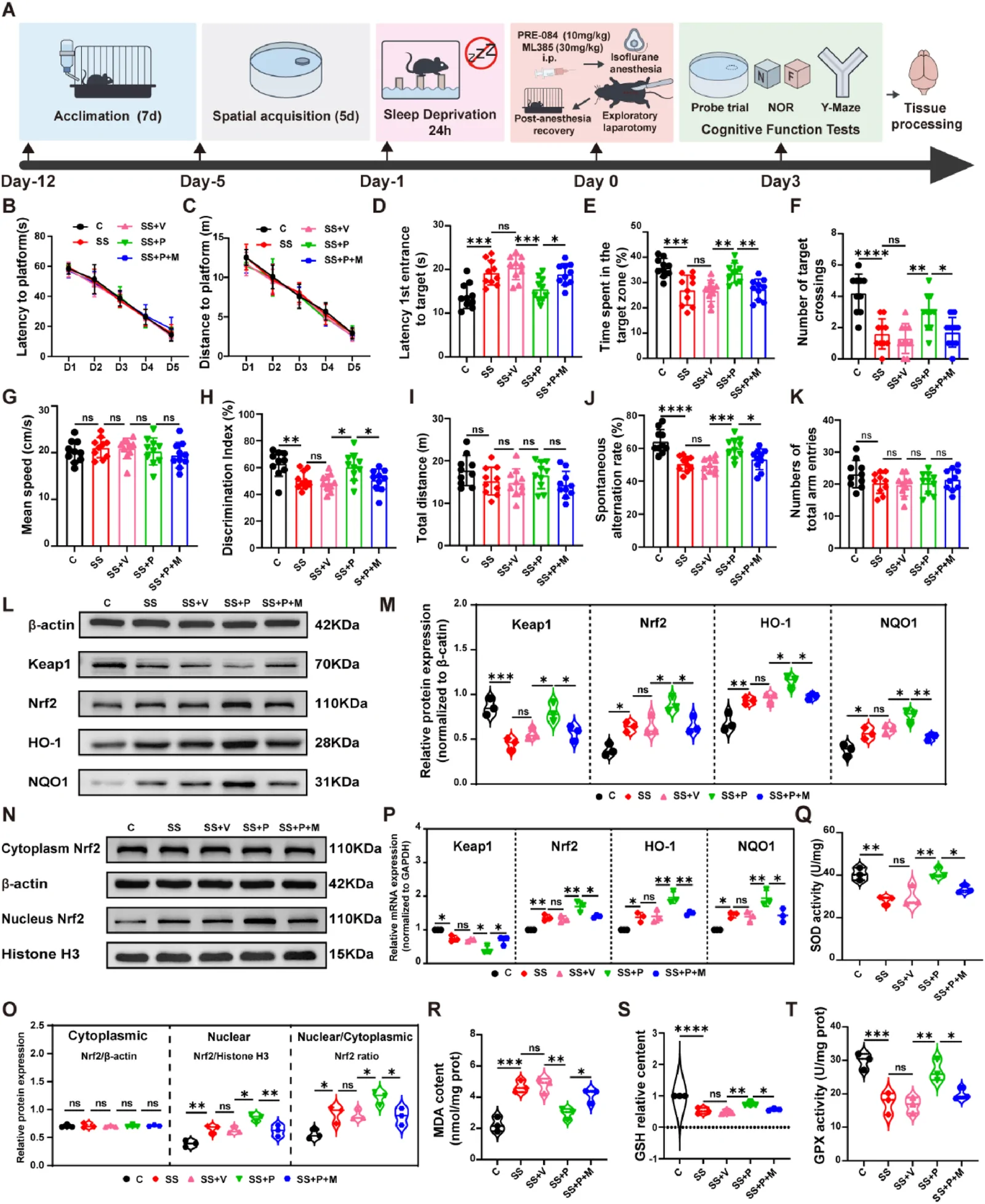
**The neuroprotective effect of σ-1R agonist is mediated via the Keap1/Nrf2 signaling pathway through anti-inflammatory and anti-oxidative actions.** (A) Schematic of the experimental timeline. Mice were acclimated for 7 days and pre-trained in the MWM for 5 days. Following 24 h of preoperative sleep deprivation, mice were intraperitoneally injected with the Nrf2 inhibitor ML385 (30 mg/kg) 2 h before surgery and the σ-1R agonist PRE-084 (10 mg/kg) 1 h before surgery. Laparotomy was performed under isoflurane anesthesia. Cognitive function was assessed on postoperative day 3 using the MWM spatial probe test, NOR test, and Y-maze test, followed by brain tissue collection. (B) Escape latency during MWM acquisition. (C) Path length to the platform. (D) Time to first platform crossing during the MWM probe test. (E) Percentage of time spent in the target quadrant. (F) Number of platform crossings. (G) Average swimming speed. (H) Discrimination index in the NOR test. (I) Total distance traveled in the NOR test. (J) Spontaneous alternation rate in the Y-maze. (K) Total number of arm entries. (L) Representative Western blot images of key proteins in the Keap1/Nrf2 pathway in hippocampal tissues. (M) Quantitative analysis of the relative protein levels shown in (L) (normalized to β-actin). (N) Representative Western blot images of nuclear and cytoplasmic Nrf2. (O) Quantitative analysis of cytoplasmic Nrf2/β-actin, nuclear Nrf2/Histone H3, and Nrf2 nuclear-to-cytoplasmic (N/C) ratio. (P) Quantitative analysis of the relative mRNA levels of key factors in the Keap1/Nrf2 pathway (normalized to GAPDH). (Q) SOD activity in hippocampal tissues. (R) MDA content. (S) GPX activity. (T) Relative GSH content. Data are presented as the mean ± SD; n = 3 (biological replicates). Statistical significance was determined by one- or two-way ANOVA with Tukey’s post hoc test. ^ns^*P* > 0.05, ∗*P* < 0.05, ∗∗*P* < 0.01, ∗∗∗*P* < 0.001, ∗∗∗∗*P* < 0.0001.

WBand RT-qPCR results demonstrated that PRE 084 significantly upregulated the expression of total Nrf2, promoted its nuclear translocation (increased nuclear Nrf2 and Nuclear/Cytoplasmic ratio), and upregulated downstream antioxidant enzymes (HO-1, NQO-1) in the hippocampus, while down-regulating Keap1. Validation of fraction purity confirmed the successful separation of cytoplasmic and nuclear proteins (Fig. S3E). ML385 effectively blocked these effects, returning the protein and mRNA levels to the pathological state (Fig. 5L–P). Regarding oxidative stress markers, PRE-084 markedly decreased MDA content, restored GSH level, and enhanced SOD and GPX activities, whereas ML385 reversed these improvements (Fig. 5Q–T). In addition, assays of inflammatory cytokines revealed that PRE-084 restored the balance of inflammation (down-regulating IL-1β, IL-6, and TNF-α; up-regulating IL-4 and IL-10), an effect that was abolished by ML385 (Fig. S3F).

These results indicate that inhibiting Nrf2 reverses the beneficial effects of the σ-1R agonist PRE-084 on postoperative cognitive function, neuroinflammation, and oxidative stress in sleep-deprived mice. This suggests that the neuroprotective action of PRE-084 is likely mediated through modulation of the Keap1/Nrf2 signaling pathway.

### The σ-1R agonist alleviates LPS-induced oxidative stress and inflammatory responses in BV2 cells by regulating the Keap1/Nrf2 pathway

To verify the regulatory effect of σ-1R on the Nrf2 pathway at the cellular level, we employed a LPS-induced BV2 microglial cell model combined with Nrf2-siRNA knockdown for mechanistic validation. CCK-8 screening identified 10 μM PRE-084 pretreatment for 30 min as the optimal treatment condition (Fig. 6B–D), and Nrf2-siRNA effectively knocked down Nrf2 expression (Fig. 6E–G). ICC results showed that LPS induction increased the expression of σ-1R and Nrf2 and promoted Nrf2 nuclear translocation; PRE-084 further enhanced Nrf2 nuclear translocation. While Nrf2 knockdown did not affect σ-1R expression, it blocked the PRE-084-induced Nrf2 nuclear translocation (Fig. 6H–J).

**Fig. 6:**
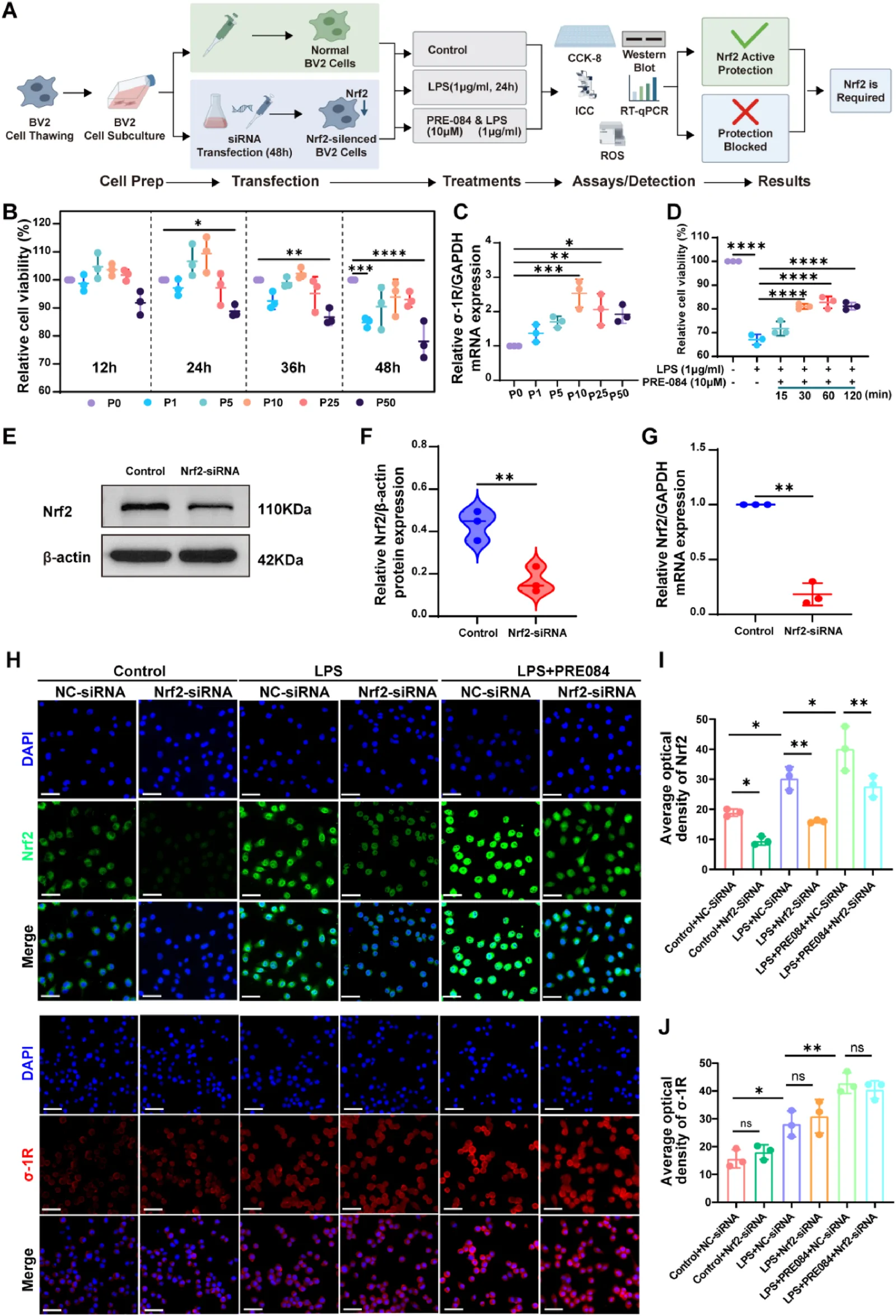
**PRE-084 effectively acts on BV2 cells and activates the Nrf2 signaling pathway.** (A) Experimental scheme in vitro: BV2 microglial cells were cultured, transfected with siRNA to establish an Nrf2-knockdown cell line, stimulated with LPS to induce injury, and pretreated (or not) with the σ-1R agonist PRE-084. Cell viability was then tested by CCK-8 assay, oxidative stress was assessed by ICC and flow cytometry, and expression of Nrf2 and related targets was detected by Western blot and RT-qPCR, confirming that Nrf2 is essential for the neuroprotection mediated by σ-1R activation. (B) Dose- and time-dependent effects of PRE-084 on BV2 cell viability (CCK-8). (C) σ-1R mRNA levels after PRE-084 treatment (RT-qPCR). (D) Cell viability after 24 h LPS (1 μg/mL) induction with different PRE-084 pretreatment times (CCK-8). (E) Representative Western blot images of Nrf2. (F, G) Quantitative analysis of Nrf2 protein (normalized to β-actin) and mRNA (normalized to GAPDH) expression. (H) ICC staining images of BV2 cells: nuclei (blue, DAPI), σ-1R (red), Nrf2 (green). Scale bar = 50 μm. (I, J) Quantitative analysis of mean fluorescence intensity for σ-1R and Nrf2. Data are presented as mean ± SD; n = 3. Statistical significance was determined by one-way ANOVA with Tukey’s post hoc test. ^ns^*P* > 0.05, ∗*P* < 0.05, ∗∗*P* < 0.01, ∗∗∗*P* < 0.001, ∗∗∗∗*P* < 0.0001.

Functional experiments confirmed that the anti-inflammatory and anti-oxidative effects of the σ-1R agonist depend on the presence of Nrf2. In NC-siRNA cells, PRE-084 significantly up-regulated the expression of Nrf2 and the downstream enzymes HO-1 and NQO-1, while down-regulating Keap1 levels (Fig. 7A–C). It also effectively suppressed the LPS-induced ROS burst and MDA production, and restored GSH content as well as SOD and GPX activities (Fig. 7D–K). However, in Nrf2-knockdown cells, the activating effect of PRE-084 on the Keap1/Nrf2 pathway was blocked, and it failed to inhibit ROS accumulation and oxidative stress damage (Fig. 7A–K). Regarding inflammatory regulation, PRE-084 restored the inflammatory balance disrupted by LPS (down-regulating IL-1β, IL-6, and TNF-α; up-regulating IL-4 and IL-10), and this anti-inflammatory effect was reversed by Nrf2 knockdown (Fig. S4).

**Fig. 7:**
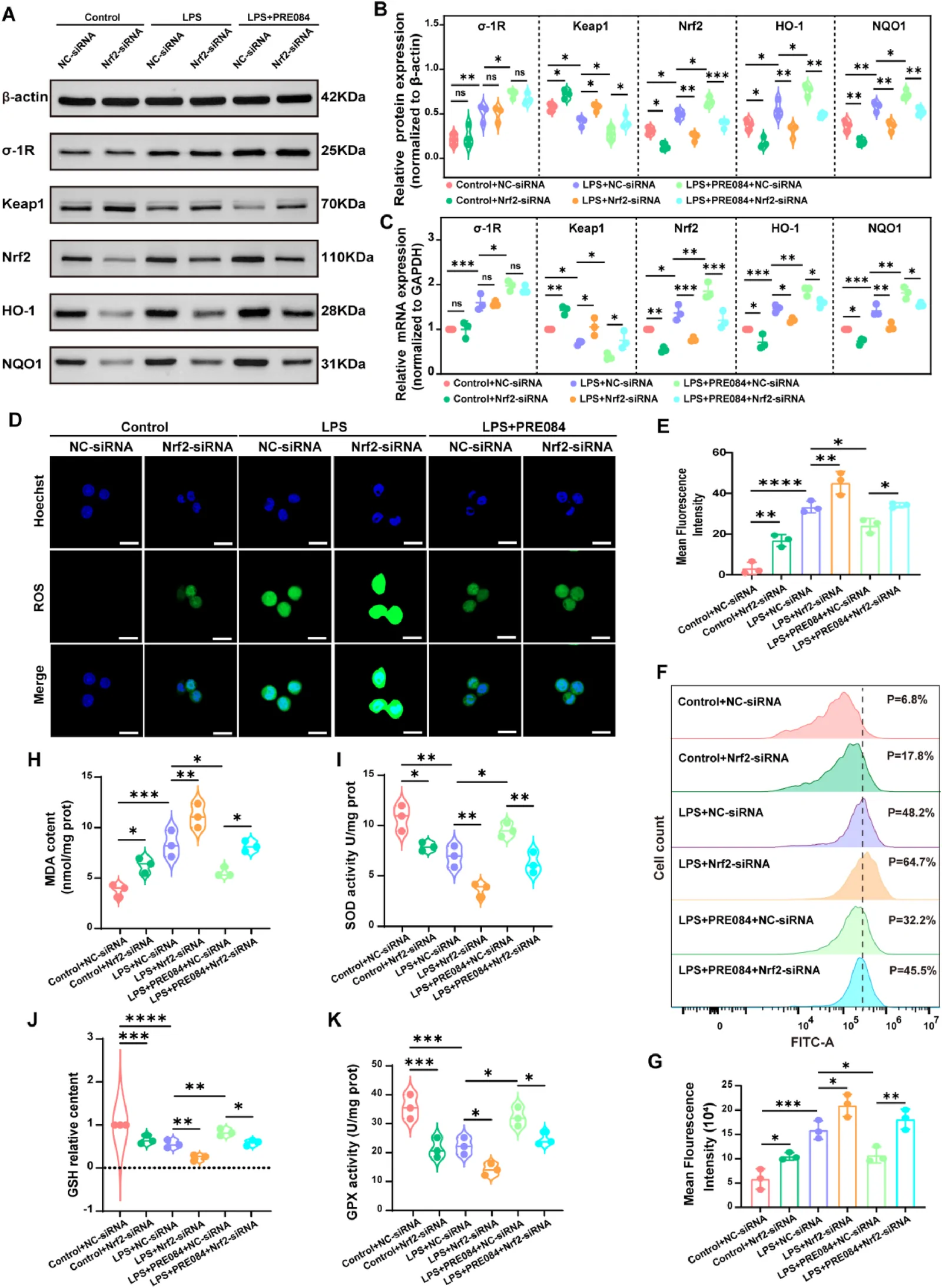
**PRE-084 alleviates LPS-induced oxidative stress and inflammatory responses in BV2 cells via an Nrf2-dependent mechanism.** (A) Representative WB images of key factors in the Keap1/Nrf2 pathway in BV2 cells from each group. (B) Quantitative analysis of the relative protein expression levels of the above factors (normalized to β-actin). (C) Quantitative analysis of the relative mRNA expression levels of the above factors (normalized to GAPDH). (D) Representative confocal microscopy images of cells from each group labeled with the DCFH-DA probe. Green fluorescence represents intracellular ROS levels; blue fluorescence (Hoechst) indicates nuclei. Scale bar = 20 μm. (E) Quantitative analysis of the mean fluorescence intensity of ROS. (F) The percentage of ROS-positive cells in each group. (G) Quantitative analysis of the mean ROS fluorescence intensity measured by flow cytometry. (H) MDA content in BV2 cells. (I) SOD activity. (J)Relative GSH content . (K) GPX activity. Data are presented as the mean ± SD; n = 3 (biological replicates). Statistical significance was determined by one-way ANOVA with Tukey’s post hoc test. ^ns^*P* > 0.05, ∗*P* < 0.05, ∗∗*P* < 0.01, ∗∗∗*P* < 0.001, ∗∗∗∗*P* < 0.0001.

In summary, this study demonstrates that the anti-inflammatory and anti-oxidative effects of the σ-1R agonist are likely achieved by modulating the Keap1/Nrf2 signaling pathway, with Nrf2 serving as a core mediator in this regulatory process.

### Dex exerts neuroprotective effects with involvement of σ-1R signaling

To evaluate the translational potential of σ-1R, we further investigated whether Dex improves postoperative cognitive impairment exacerbated by sleep deprivation and its association with σ-1R. PBPK modeling predicted favorable blood-brain barrier penetration by Dex, with a high predicted tissue-to-plasma partition coefficient in the brain (47.55), which was significantly higher than that in other peripheral tissues (Fig. 8A), suggesting effective brain exposure. Molecular docking revealed that Dex stably occupies the ligand-binding pocket of σ-1R (binding energy: 7.5 kcal/mol), mainly through hydrogen bonding, hydrophobic interactions, and π-π stacking, with key residues including Val84, Trp89, Met93, Leu95, Tyr103, Leu105, Phe107, Ile124, and Trp164 (Fig. 8B).

**Fig. 8:**
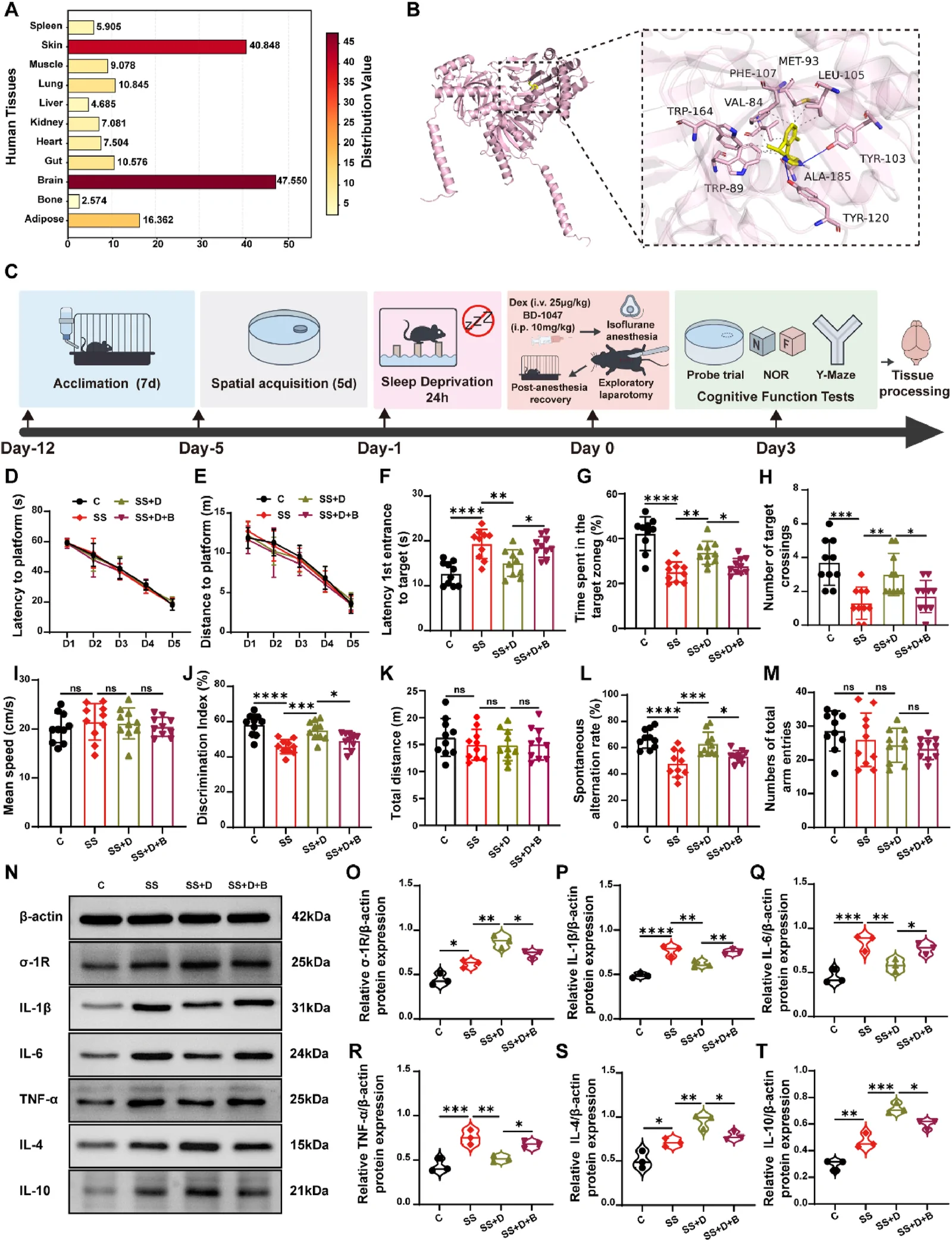
**Dex alleviates postoperative cognitive impairment exacerbated by preoperative sleep deprivation through σ-1R activation.** (A) Predicted tissue distribution of Dex in a standard human PBPK model. (B) Molecular docking simulation of Dex within the ligand-binding pocket of σ-1R (gray surface). A close-up view of the binding site is shown in the right panel, with key interacting residues labeled. (C) Experimental timeline: mice were acclimated for 7 days and pre-trained in the MWM for 5 days. After 24 h of preoperative sleep deprivation, they received intraperitoneal injection of the σ-1R agonist PRE-084 (10 mg/kg, 1 h before surgery) and intravenous injection of DEX (25 μg/kg, 30 min before surgery). Laparotomy was performed under isoflurane anesthesia. Cognitive function was assessed on postoperative day 3 using the MWM spatial probe test, NOR test, and Y-maze test, followed by brain tissue collection. (D) Escape latency and (E) Path length to the platform during MWM acquisition (n = 10 per group). (F) Time to first platform crossing, (G) Percentage of time in the target quadrant, (H) Number of platform crossings, and (I) Average swimming speed during the MWM probe test (n = 10 per group). (J) Discrimination index and (K) total distance traveled in the NOR test (n = 10 per group). (L) Spontaneous alternation rate and (M) Total arm entries in the Y-maze (n = 10 per group). (N) Representative WB images of σ-1R and inflammatory cytokines (IL-1β, IL-6, TNF-α, IL-4, IL-10) in hippocampal tissues (n = 3 per group). (O) Quantitative analysis of the relative protein expression levels (normalized to β-actin). Data are presented as the mean ± SD. Statistical significance was determined by one-way ANOVA with Tukey’s post hoc test. ^ns^*P* > 0.05, ∗*P* < 0.05, ∗∗*P* < 0.01, ∗∗∗*P* < 0.001, ∗∗∗∗*P* < 0.0001.

Behavioral assessments showed that Dex significantly alleviated cognitive deficits in model mice. In the MWM, Dex shortened the latency to first platform crossing, increased the time spent in the target quadrant, and raised the number of platform crossings (Fig. 8D–I and Fig. S5A and B). In the NOR and Y-maze tests, Dex significantly prolonged novel-object exploration time and improved the spontaneous alternation rate (Fig. 8J–M and Fig. S5C and D). Importantly, the σ-1R antagonist BD-1047 reversed these cognitive benefits of Dex. At the molecular level, Dex treatment significantly up-regulated σ-1R protein and mRNA expression in the hippocampus, promoted the production of anti-inflammatory cytokines, suppressed pro-inflammatory factors(Fig. 8N-T). Furthermore, Dex modulated the Keap1/Nrf2 signaling pathway, as evidenced by decreased Keap1 mRNA levels and increased mRNA expression of Nrf2 and its downstream targets HO-1 and NQO1(Fig. S5E and F). All these molecular changes were abolished by co-administration of BD 1047, confirming that Dex acts through σ-1R to regulate neuroinflammation and the Keap1/Nrf2 axis. .

In summary, leveraging its excellent brain penetrability, Dex significantly ameliorated POCD exacerbated by preoperative sleep deprivation, likely through, at least in part, engaging σ-1R signaling and modulating downstream anti-inflammatory and anti-oxidative pathways.

## Discussion

In this study, we have demonstrated for the first time that in a model of POCD exacerbated by preoperative sleep deprivation, σ-1R is upregulated in the hippocampus. Furthermore, pharmacological activation of σ-1R with PRE-084 significantly improved cognitive function, which was accompanied by suppression of microglial activation-associated neuroinflammation and oxidative stress, likely through modulating the Keap1/Nrf2 signaling axis. Additionally, we found that one of the key mechanisms by which Dex exerts neuroprotective effects involves its activation of σ-1R (Fig. 9).

**Fig. 9:**
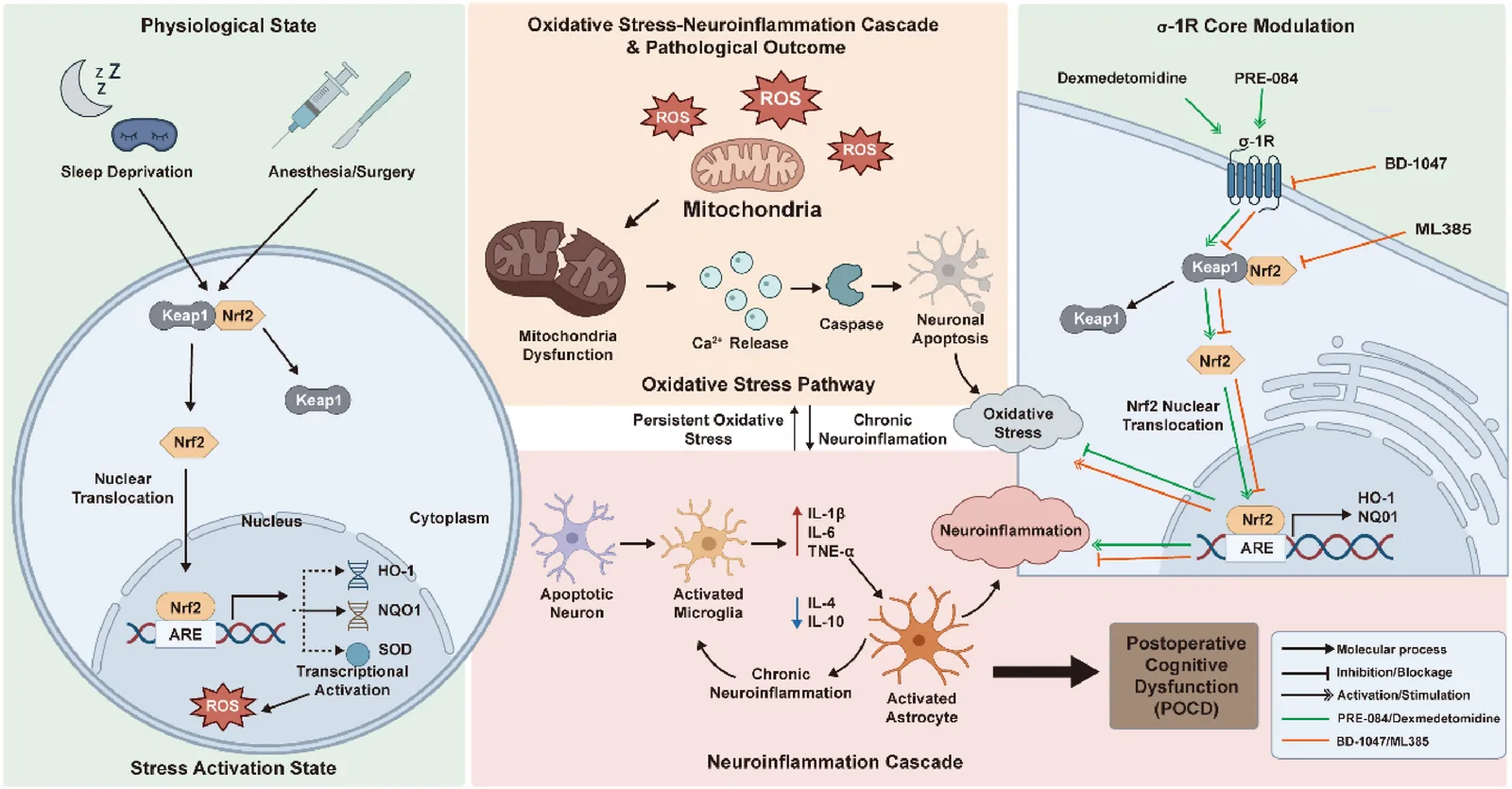
**The crosstalk between σ-1R and the Keap1/Nrf2 signaling axis: Targeting neuroinflammation and oxidative stress to alleviate postoperative cognitive impairment exacerbated by preoperative sleep deprivation.** Left panel: Under physiological conditions, Nrf2 is bound by Keap1 and sequestered in the cytoplasm. Under oxidative stress, Nrf2 dissociates from Keap1, translocates into the nucleus, and initiates the transcription of downstream antioxidant genes. Middle panel: Preoperative sleep deprivation and surgical trauma induce mitochondrial dysfunction and calcium overload, leading to microglial activation. This results in the release of pro-inflammatory cytokines, neuronal apoptosis, and exacerbation of cognitive impairment via a vicious cycle of “oxidative stress-neuroinflammation.” Right panel: Pharmacological modulation of σ-1R. The agonist PRE-084 and the clinical drug Dex activate the antioxidant pathway by promoting Nrf2 nuclear translocation, whereas the antagonist BD-1047 blocks this effect. Additionally, the Nrf2 inhibitor ML385 directly inhibits downstream signaling. Symbol Legend: Simple arrows indicate molecular processes; double arrows indicate promotion or activation; T-shaped lines indicate inhibition. Different colors are used to denote different pharmacological interventions.

POCD is a common neurological complication in the perioperative period, with an intricate and complex pathological mechanism. It is widely accepted that the core components involve an imbalance in oxidative stress, neuroinflammation, and impairment of hippocampal-dependent cognitive circuits [17], [18]. Sleep deprivation is a prevalent clinical stressor. Prolonged sleep deprivation has been demonstrated to lead to progressive cognitive decline by impairing hippocampal neuronal plasticity, inducing systemic inflammation, and accumulating oxidative damage [15], [19]. However, whether short-term sleep deprivation acts synergistically with surgical stress to exacerbate postoperative cognitive impairment remains a subject of debate [20], [21]. This study observed that while 24 h of sleep deprivation alone was insufficient to directly induce cognitive deficits, it increased the risk of POCD by exacerbating the perioperative stress burden and redox imbalance, thereby weakening the brain’s functional compensatory capacity. This finding is highly consistent with the clinical phenomenon wherein patients with preoperative sleep disorders exhibit a higher incidence and more severe symptoms of postoperative cognitive complications [22], [23]. To simulate this clinical scenario, this study established a 24 h sleep deprivation model using a modified multiple-platform water environment method, which was combined with exploratory laparotomy to construct a dual-stress POCD model. This model demonstrated clear deficits in learning and memory abilities at the behavioral level (Fig. 1A-O), not only retaining the pathological basis of the single surgical stress model but also highlighting the synergistic exacerbating effect of sleep deprivation on surgical stress. Compared to traditional single-factor models, this dual-stress model more closely approximates the actual clinical setting of multiple perioperative stressors, providing a more reliable and physiologically relevant experimental platform for deciphering the mechanisms of POCD under complex conditions. This finding also suggests that even for patients with good surgical tolerance, optimizing preoperative sleep status and alleviating perioperative redox pressure may still be key intervention points for reducing the risk and severity of POCD. It is important to specifically note that in clinical practice, anesthesia and surgery are inseparable, together constituting the core stress factors of the perioperative period. Although anesthesia itself is also a significant contributor to POCD, to maintain alignment with the actual clinical context, this study did not separate their effects; the term “surgery” used in the text refers to the combined synergistic action of anesthesia and surgery.

σ-1R, a highly expressed “stress sensor” in the central nervous system, demonstrates changes in its expression and distribution that are closely linked to neural damage and repair processes, as evidenced in models such as traumatic brain injury [24], [25]. Our study further corroborates this conclusion within the dual-stress POCD model, revealing that the compensatory upregulation of σ-1R is specific to the hippocampus with no changes observed in the prefrontal cortex, indicating the hippocampus as the core brain region for σ-1R-mediated cognitive regulation (Fig. 2A–C, E and Fig. S2A–D). This finding aligns with literature reports on the significant regulatory role of σ-1R in brain regions critical for learning and memory [26], [27]. Within the CNS, σ-1R is widely expressed in neurons and all glial cell types [28]. Our findings indicate that under normal conditions, σ-1R is primarily expressed in neurons, with lower levels in astrocytes and minimal expression in microglia. In the POCD model, however, σ-1R was significantly upregulated in all three cell types, with the most pronounced inducible change observed in microglia (Fig. 2A–D). Given that excessive microglial activation is a central driver of neuroinflammation and oxidative stress in POCD [29], the prominent inducible expression of σ-1R in these cells suggests its direct involvement in regulating microglial immune responses, a hypothesis validated in subsequent BV2 microglial cell experiments. Pharmacological experiments support a role for σ-1R in this process. The agonist PRE-084 effectively reversed stress-induced cognitive deficits, whereas the antagonist BD-1047 exacerbated cognitive damage, demonstrating a bidirectional effect consistent with σ-1R involvement (Fig. 3A-O). At first glance, this appears paradoxical: the endogenous σ-1R was already elevated in the model group, yet further increasing it with PRE-084 improved cognition, while decreasing it with BD-1047 worsened the deficits. This apparent contradiction is resolved by recognizing that the endogenous upregulation represents a compensatory but insufficient protective response, which can be therapeutically amplified by exogenous PRE-084. Interestingly, we found that the expression of σ-1R itself is bidirectionally regulated by its ligands. In the SS group, where σ-1R levels were already compensatorily elevated, the agonist PRE-084 further increased its protein and mRNA expression, while the inhibitor BD-1047 significantly reduced it (Fig. 4H–J). We attribute this bidirectional regulation to the unique property of σ-1R as an ER-resident molecular chaperone. Under pathological stress, σ-1R undergoes compensatory transcriptional upregulation via the PERK/eIF2α/ATF4 pathway to maintain proteostasis [30]. The exogenous agonist PRE-084 may act as a molecular stabilizer, potentially enhancing its interaction with chaperone proteins (e.g., Binding immunoglobulin protein) by promoting receptor conformational changes, thereby improving its anti-ER stress capacity and protein stability [31]. Conversely, the inhibitor BD-1047, while occupying the binding site, fails to induce a protective conformation. It not only blocks downstream signaling but may also trigger a negative feedback loop, leading to an overall reduction in receptor levels [32]. In summary, these findings indicate that the dynamic plasticity of σ-1R is fundamental to its rapid response to acute stress and its role in facilitating neural repair.

The Keap1/Nrf2 pathway, as the core defense system against oxidation and inflammation in the body, plays a crucial role in σ-1R-mediated neuroprotection, which has formed a broad consensus in various models of neurological injury [33], [34]. However, its precise regulatory mechanism in the context of POCD remains to be clarified. In the dual-stress POCD model of this study, we observed downregulated expression of Keap1 alongside upregulated levels of Nrf2 (including both total and nuclear protein) and its downstream target genes HO-1 and NQO1 in the mouse hippocampus, indicating activation of the endogenous defense pathway (Fig. 5L-P). This finding is consistent with the inherent compensatory response of the organism to surgical stress [35]. However, this endogenous activation proved insufficient to fully counterbalance the intense perioperative stress: despite the induction of the Nrf2 pathway, markers of oxidative stress (MDA, ROS) remained significantly elevated in hippocampal tissue, while the activities of key antioxidant enzymes (SOD, GPX) and GSH content were markedly reduced (Fig. 5Q-T). This paradoxical phenomenon of “pathway activation with persistent oxidative imbalance” has also been reported in models of cerebral ischemia-reperfusion injury [36], suggesting that excessive stress load may ultimately overwhelm the compensatory threshold of the endogenous antioxidant system. A key finding of this study is that exogenous administration of the σ-1R agonist PRE-084 effectively resolves this contradiction. PRE-084 intervention not only synergistically enhanced Nrf2 nuclear translocation, downstream target gene expression, and downregulated Keap1, but more importantly, it concurrently reversed the aforementioned abnormalities in oxidative stress and inflammatory markers, achieving a restoration of redox and inflammatory homeostasis (Fig. 5L-T and Fig. S3F). The Nrf2-dependence of this protective effect was validated both in vivo and in vitro: in vivo, the Nrf2-specific inhibitor ML385 reversed the beneficial effects of PRE-084, a phenomenon consistent with findings in other organ injury models [37]; in vitro, knockdown of Nrf2 in BV2 microglial cells similarly significantly attenuated the protective effects of PRE-084 (Fig. 7A–K). To further elucidate the direct molecular mechanism by which σ-1R regulates Nrf2, we found (Fig. 4K and L) that in the hippocampal tissue of the POCD model, the co-localization signal between σ-1R and Nrf2 was enhanced, and PRE-084 intervention further promoted their co-localization and Nrf2 nuclear translocation. Subsequent Co-IP experiments confirmed (Fig. 4M) that, while being accompanied by reduced Nrf2 association with Keap1, σ-1R may associate with Keap1, suggesting a more refined regulatory mode: σ-1R might modulate the Keap1-Nrf2 interaction through competitive or allosteric modulation, thereby facilitating Nrf2 stabilization and nuclear translocation.

To date, no definitive and effective pharmacotherapeutic agents are available in clinical practice for preventing or treating POCD. Although evidence suggests that certain perioperative medications, such as Dex, lidocaine, and esketamine, hold preventive potential [38], [39], [40], their precise mechanisms, and especially their efficacy in patients with comorbid conditions like sleep disorders, require further in-depth exploration. A key finding of this study is that the neuroprotective effect of Dex could be partially blocked by the σ-1R antagonist (Fig. 8D-T). This indicates that, in addition to its classic action as an α2-adrenergic receptor agonist [41], engaging σ-1R signaling represents one pathway through which Dex may exert its neuroprotective effects. This discovery not only reveals a new dimension of the multi-target pharmacological mechanism of Dex but also provides direct experimental evidence for its more precise clinical application in specific high-risk populations, such as those with comorbid sleep disorders.

Although this study has achieved clear mechanistic breakthroughs, certain limitations remain. First, the present study relies exclusively on pharmacological approaches (PRE-084 and BD-1047) to implicate σ-1R in the observed effects. Although these compounds are well-characterized and widely used in the field, pharmacological agents can exhibit off-target actions. Future studies employing genetic approaches, such as Sigma1 knockdown or knockout, would provide more definitive evidence for receptor-specific effects and further strengthen the conclusions drawn here. Second, it did not explore the crosstalk regulation between σ-1R and other signaling pathways (such as MAPK and PI3K/Akt), which may represent an important complementary aspect of its multi-dimensional protective effects. In the future, we will further dissect the detailed regulatory network mediated by σ-1R as a core target. Third, the protective effects of the σ-1R agonist were validated only at the animal and cellular levels in this study, lacking verification with clinical samples. Future work should involve collecting cerebrospinal fluid or brain tissue samples from POCD patients to examine expression changes in the σ-1R/Nrf2 pathway, thereby providing direct evidence for clinical application.

This study establishes the critical neuroprotective role of the σ-1R/Keap1/Nrf2 signaling axis in alleviating POCD exacerbated by preoperative sleep deprivation. Activation of σ-1R can inhibit microglia-associated neuroinflammation and restore redox homeostasis, thereby rescuing cognitive deficits. Importantly, we found that σ-1R signaling may partially contribute to the brain-protective effects of Dex, thereby bridging a preclinical mechanistic discovery with an established clinical pharmacotherapy. These findings not only provide a mechanistic explanation for the high susceptibility to postoperative cognitive decline in patients with sleep disorders but also identify σ-1R as a translational target for precise perioperative neuroprotection in vulnerable surgical populations, holding significant scientific value and clinical potential.

## CRediT authorship contribution statement

Xiuhua Li: Conceptualization, Methodology, Investigation, Data curation, Writing – original draft. Baorui Guo: Data curation, Investigation, Methodology, Validation, Funding acquisition. Jiatai An: Investigation, Methodology. Yuansong Wei: Investigation, Methodology. Jin Yu: Supervision, Validation, Writing – review & editing. Peng Li: Supervision, Validation, Writing – review & editing. Aiping Zheng: Supervision. Liang Xu: Conceptualization, Supervision, Project administration, Funding acquisition, Writing – review & editing. Yalan Li: Conceptualization, Supervision, Project administration, Funding acquisition, Writing – review & editing.

## Funding

This work was supported by the Guangzhou Science and Technology Project [grant No. 2023A03J1019]; the 10.13039/501100004826Beijing Natural Science Foundation [grant No. L244039L244039] and the Shaanxi Provincial Natural Science Basic Research Program [Grant No. 2024JC-YBQN-0952].

## Declaration of competing interests

We confirm that we have no financial or personal affiliations with any individuals or organizations that might unduly affect our work. Additionally, there are no professional or personal interests of any sort in relation to any product, service, or company that could be interpreted as biasing the views expressed or the evaluation contained within this manuscript.

## Figures and Tables

**figs1:**
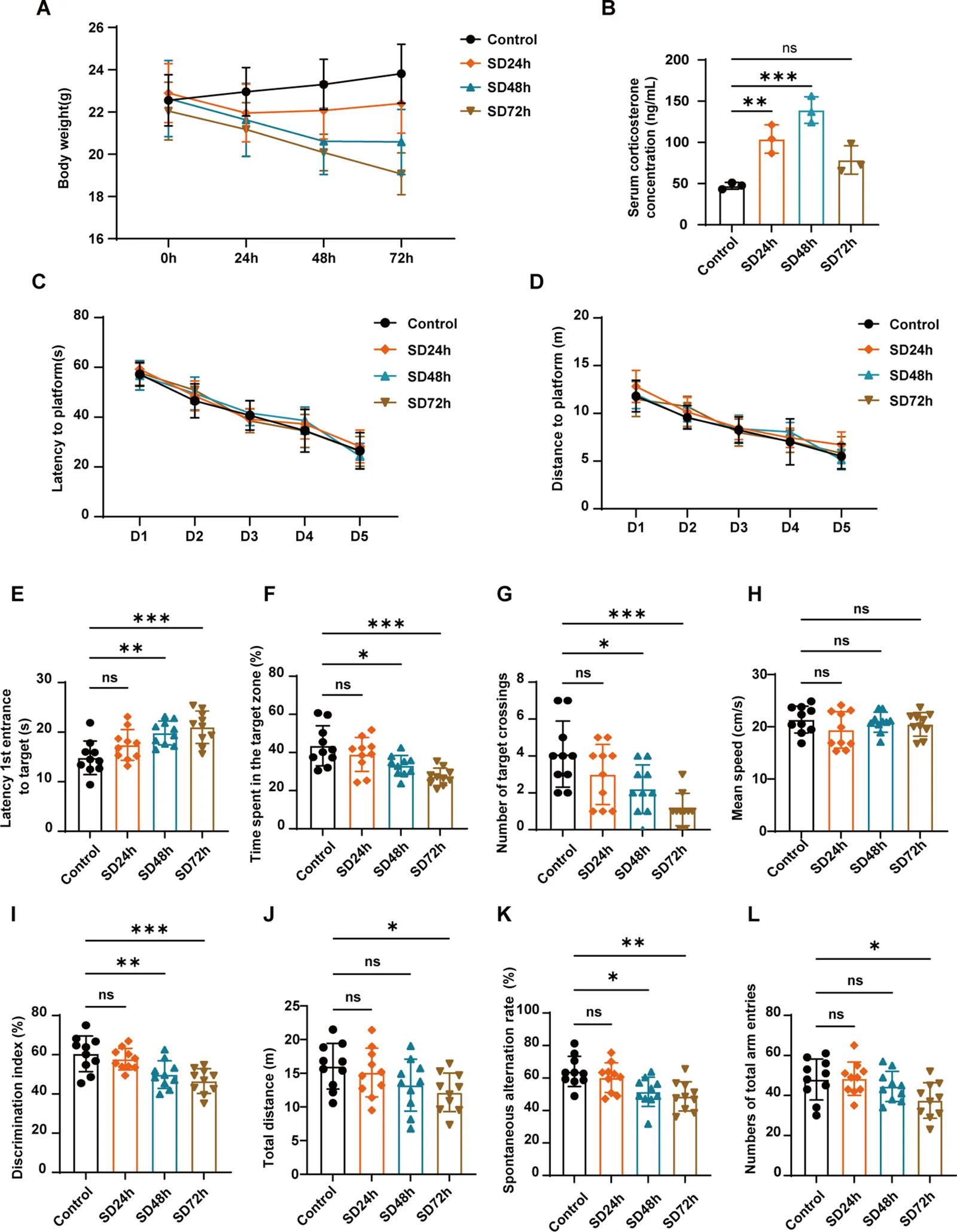


**figs2:**
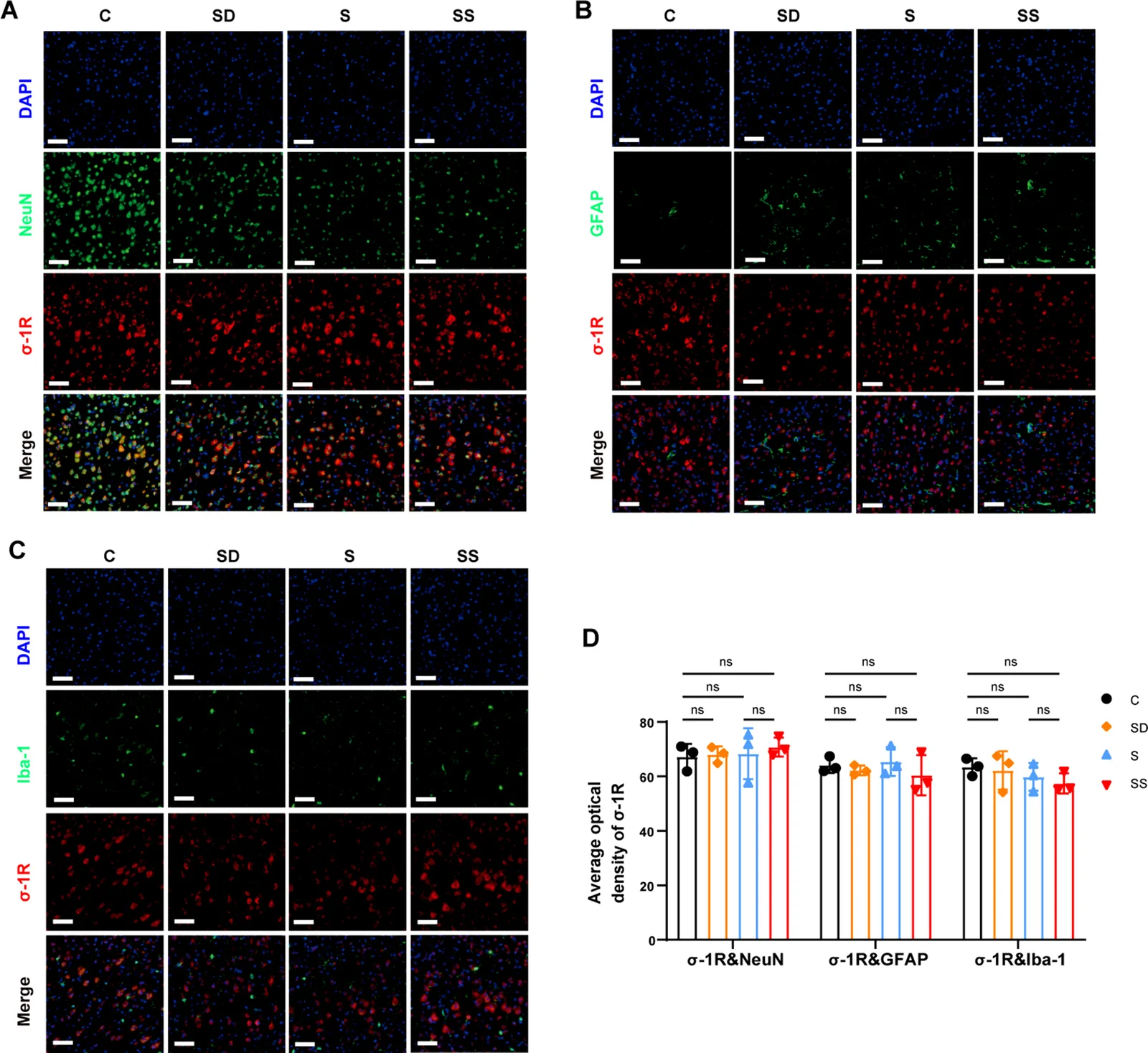


**figs3:**
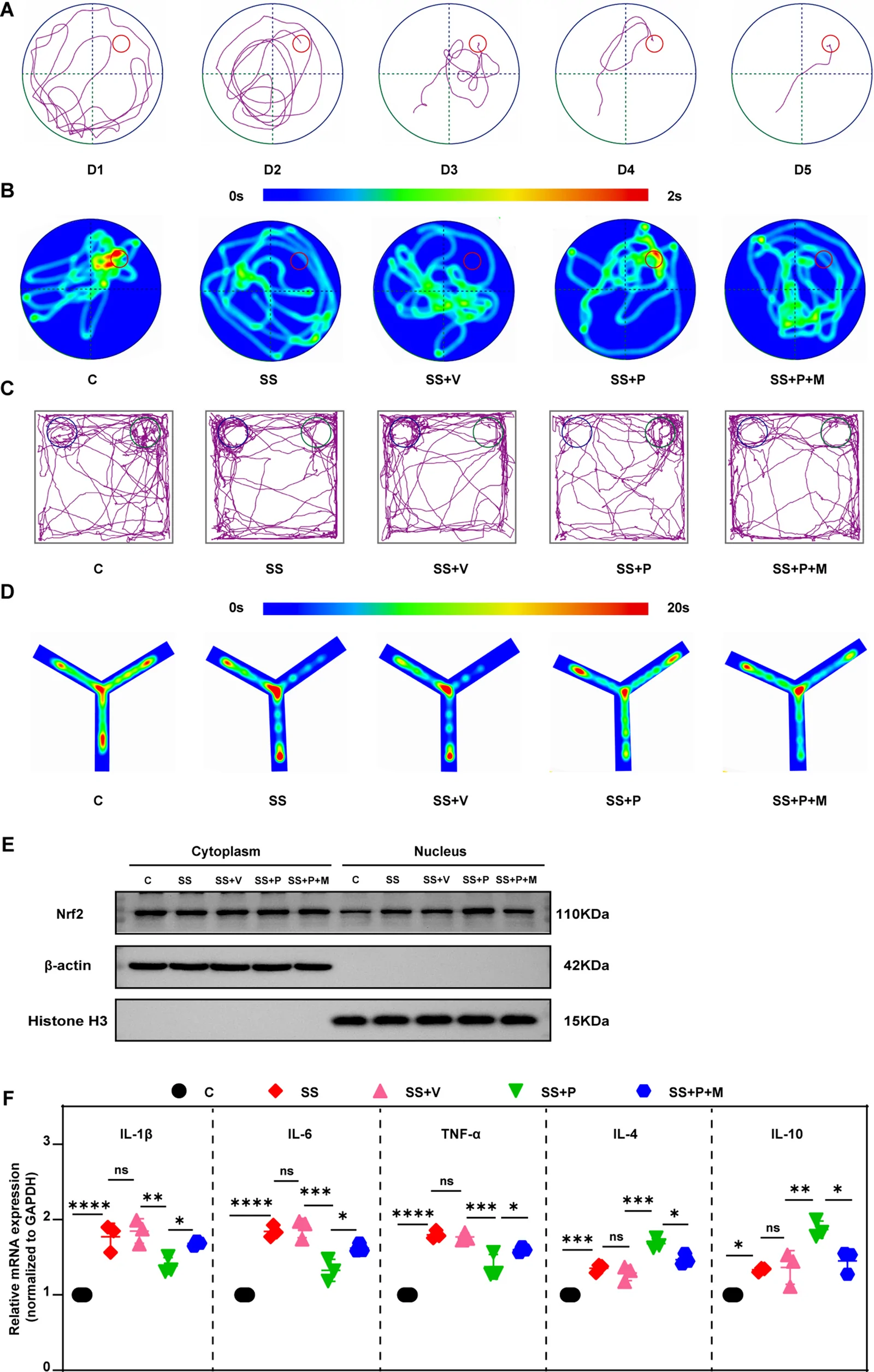


**figs4:**
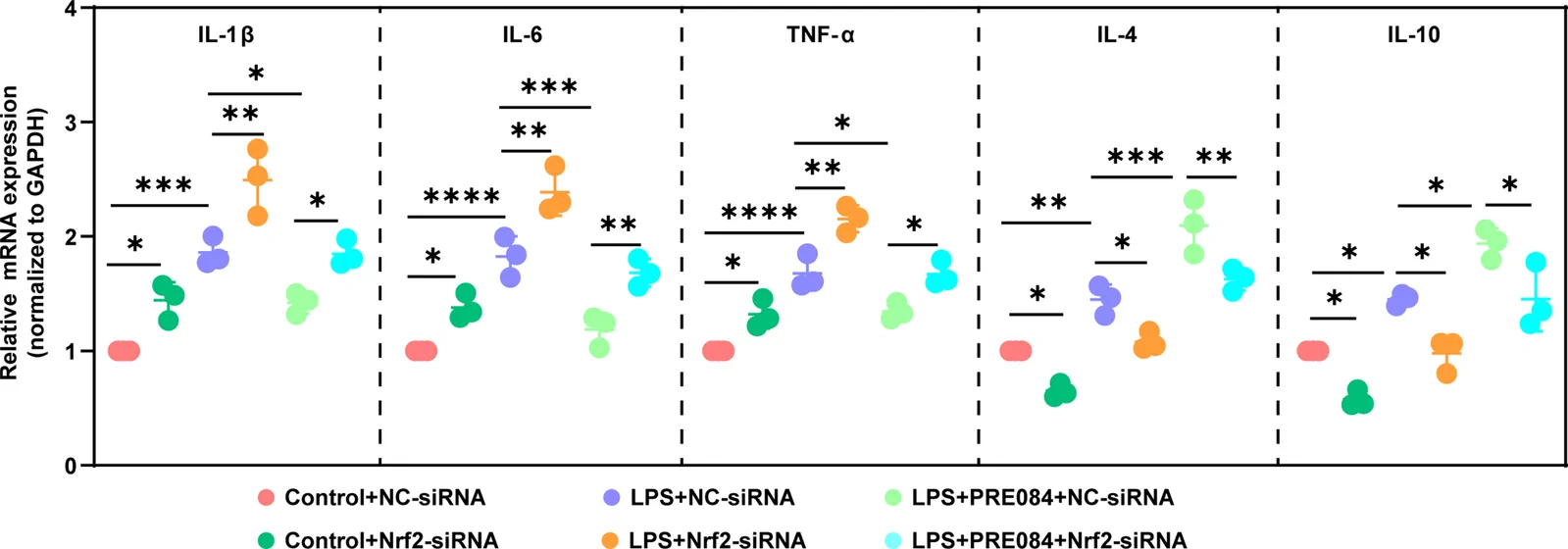


**figs5:**
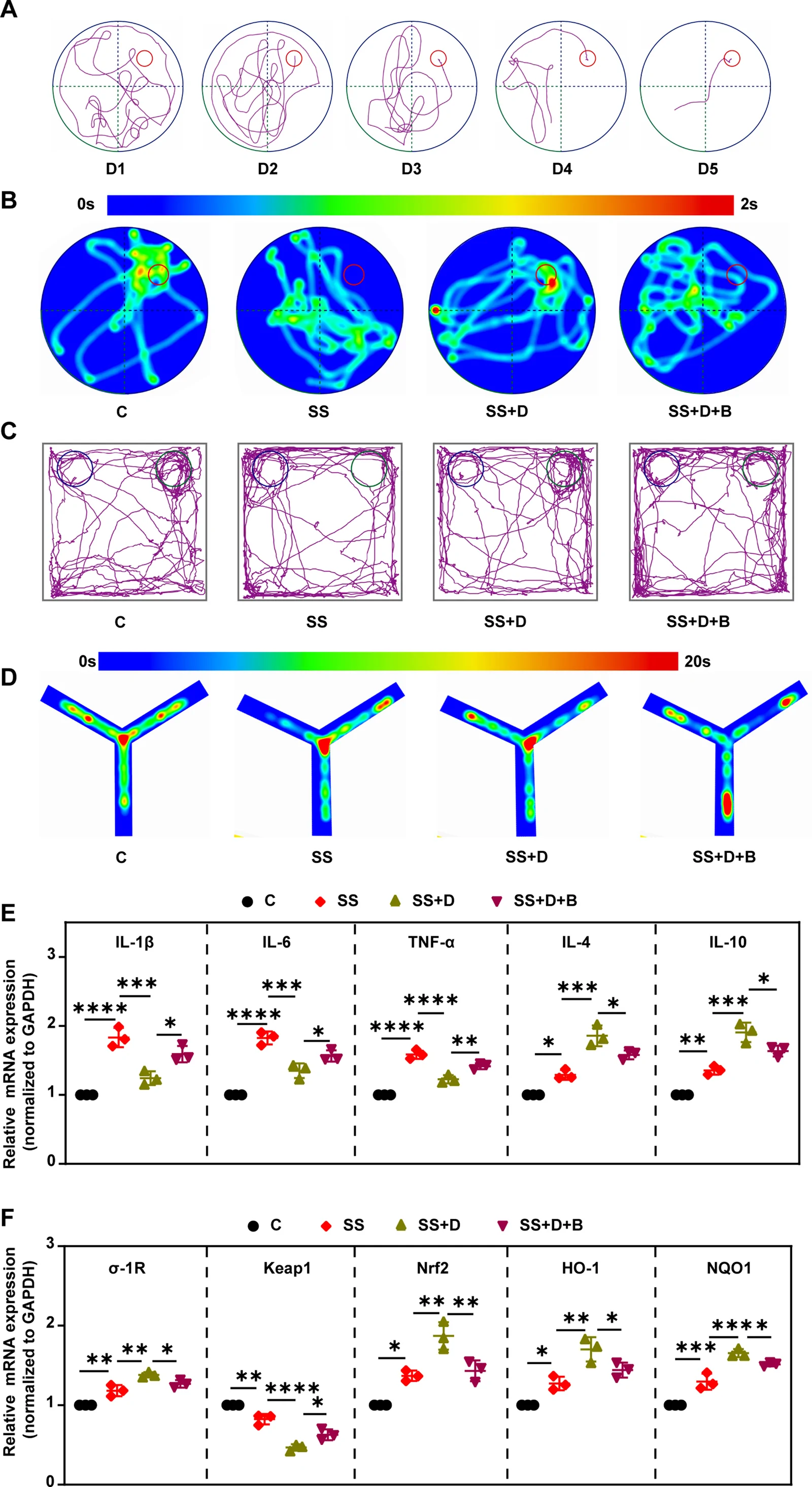


## Abbreviation

**Abbreviation, Full Name**
ANOVA: Analysis of variance
BCA: Bicinchoninic acid
CA1: Cornu Ammonis 1
CCK-8: Cell counting kit-8
Co-IP: Co-immunoprecipitation
cDNA: Complementary DNA
DAPI: 4′,6-diamidino-2-phenylindole
DCFH-DA: 2′,7′-dichlorodihydrofluorescein diacetate
Dex: Dexmedetomidine
GAPDH: Glyceraldehyde 3-phosphate dehydrogenase
GPX: Glutathione peroxidase
GSH: Glutathione
HO-1: Heme oxygenase-1
IF: Immunofluorescence
ICC: Immunocytochemistry
Keap1: Kelch-like ECH-associated protein 1
MAPK: Mitogen-activated protein kinase
MDA: Malondialdehyde
MWM: Morris water maze
NQO1: NAD(P)H quinone dehydrogenase 1
NOR: Novel object recognition
Nrf2: Nuclear factor erythroid 2-related factor 2
PBS: Phosphate-buffered saline
PBPK: Physiologically based pharmacokinetics
POCD: Postoperative cognitive dysfunction
ROS: Reactive oxygen species
RT-qPCR: Reverse transcription quantitative polymerase chain reaction
SD: Standard deviation
SOD: Superoxide dismutase
σ-1R: Sigma-1 receptor
