# Supplementary material for: Sigma-1 receptor alleviates postoperative cognitive dysfunction exacerbated by preoperative acute sleep deprivation in mice via modulating the Keap1/Nrf2 signaling pathway
Source: Neurotherapeutics. 2026 Oct 29;24(1):e01109. doi: 10.1016/j.neurot.2026.e01109 (PMC13743525; doi:10.1016/j.neurot.2026.e01109)
Supplement: Supplementary file 1 [file mmc2.docx]

Supplementary Figure 1

**Fig S1 Effects of varying durations of sleep deprivation on cognitive function in mice.** (A) Body weight changes over 72 hours across Control (mice placed on a large platform), SD24h, SD48h, and SD72h groups, n=10. (B) Serum corticosterone concentrations measured by ELISA at the endpoint of sleep deprivation (cardiac blood collection), n = 3. (C) Escape latency during the acquisition phase of the MWM test. (D) Swimming distance during the acquisition phase of the MWM test. (E) Latency to first cross the platform location during the probe trial of the MWM test. (F) Percentage of time spent in the target quadrant during the probe trial of the MWM test. (G) Number of crossings over the platform location during the probe trial of the MWM test. (H) Average swimming speed during the acquisition phase of the MWM test. (I) Discrimination index in the NOR test. (J) Total exploration distance in the NOR test. (K) Spontaneous alternation percentage in the Y-maze test. (L) Total number of arm entries in the Y-maze test. Data are expressed as mean ± standard deviation (SD), with n = 10 per group. Statistical analyses were performed using one-way or two-way analysis of variance (ANOVA) followed by Tukey’s post hoc test. ^ns^*P* > 0.05, ^*^*P* < 0.05, ^**^*P* < 0.01, ^***^*P* < 0.001, ^****^*P* < 0.0001.

Supplementary Figure 2

**Fig S2 Cellular localization and expression of σ-1R in the mouse cerebral cortex.** (A-C) Representative immunofluorescence images of mouse cortical tissues. Nuclei are stained with DAPI (blue); σ-1R is shown in red; NeuN (A), GFAP (B), and Iba-1 (C) are shown in green, representing neurons, astrocytes, and microglia, respectively. (D) Quantitative analysis of the average fluorescence intensity of σ-1R co-localization with NeuN, GFAP, and Iba-1. Data are expressed as mean ± SD. For each group, three mice were analyzed; two non-overlapping fields were randomly selected from each mouse, and the values were averaged to obtain a single value per mouse for statistical analysis. Statistical significance was determined by one-way ANOVA followed by Tukey’s post hoc test. ^ns^*P* > 0.05, ^*^*P* < 0.05, ^**^*P* < 0.01, ^***^*P* < 0.001, ^****^*P* < 0.0001.

Supplementary Figure 3

**Fig S3 Behavioral trajectories and inflammatory cytokine mRNA expression in the hippocampus of mice.** (A) Representative swimming trajectories during the acquisition phase of the MWM test. (B) Representative swimming trajectories during the probe trial of the MWM test. (C) Representative exploration trajectories in the NOR test. (D) Representative arm entry trajectories in the Y-maze test. (E) Representative Western blot images of Nrf2 in hippocampal tissues, nuclear and cytoplasmic fractions were extracted from hippocampal tissue using NE-PER™ Nuclear and Cytoplasmic Extraction Reagents. (F) Quantitative analysis of relative mRNA expression levels of inflammatory cytokine. Data are expressed as mean ± SD, with n = 3 per group. Statistical significance was determined by one-way ANOVA followed by Tukey’s post hoc test. ^ns^*P* > 0.05, ^*^*P* < 0.05, ^**^*P* < 0.01, ^***^*P* < 0.001, ^****^*P* < 0.0001.

Supplementary Figure 4

**Fig S4 Effect of Nrf2 knockdown on inflammatory cytokine mRNA expression in BV2 cells.** BV2 cells were divided into two groups: control group (transfected with scramble siRNA) and Nrf2 knockdown group (transfected with Nrf2 siRNA). Cells were treated as follows: (1) untreated control; (2) stimulated with 1 μg/mL LPS for 24 h; (3) pretreated with 10 μM PRE-084 for 30 min, followed by stimulation with 1 μg/mL LPS for 24 h. Relative mRNA expression levels of inflammatory cytokines were quantified by qPCR. Data are expressed as mean ± SD, with n = 3 per group. Statistical significance was determined by one-way ANOVA followed by Tukey’s post hoc test. ^ns^*P* > 0.05, ^*^*P* < 0.05, ^**^*P* < 0.01, ^***^*P* < 0.001, ^****^*P* < 0.0001.

Supplementary Figure 5

**Fig S5 Behavioral trajectories and mRNA expression levels of related factors in the hippocampus of mice.** (A) Representative swimming trajectories during the acquisition phase of the MWM test. (B) Representative swimming trajectories during the probe trial of the MWM test. (C) Representative exploration trajectories in the NOR test. (D) Representative arm entry trajectories in the Y-maze test. (E-F) Quantitative analysis of relative mRNA expression levels of inflammatory cytokines (E) and key factors in the Nrf2 pathway (F) by qPCR. Data are expressed as mean ± SD, with n = 3 per group. Statistical significance was determined by one-way ANOVA followed by Tukey’s post hoc test. ^ns^*P* > 0.05, ^*^*P* < 0.05, ^**^*P* < 0.01, ^***^*P* < 0.001, ^****^*P* < 0.0001.
